# A systematic review and meta-analysis of the epidemiology of pathogenic *Escherichia coli* of calves and the role of calves as reservoirs for human pathogenic *E. coli*

**DOI:** 10.3389/fcimb.2015.00023

**Published:** 2015-03-12

**Authors:** Rafał Kolenda, Michał Burdukiewicz, Peter Schierack

**Affiliations:** ^1^Faculty of Natural Sciences, Brandenburg University of Technology Cottbus–SenftenbergSenftenberg, Germany; ^2^Department of Genomics, Faculty of Biotechnology, University of WrocławWrocław, Poland

**Keywords:** calves, diarrhea, ETEC, EPEC, STEC, EHEC, systematic review, meta-analysis

## Abstract

*Escherichia coli* bacteria are the most common causes of diarrhea and septicemia in calves. Moreover, calves form a major reservoir for transmission of pathogenic *E. coli* to humans. Systematic reviews and meta-analyses of publications on *E. coli* as calf pathogens and the role of calves as reservoir have not been done so far. We reviewed studies between 1951 and 2013 reporting the presence of virulence associated factors (VAFs) in calf *E. coli* and extracted the following information: year(s) and country of sampling, animal number, health status, isolate number, VAF prevalence, serotypes, diagnostic methods, and biological assays. The prevalence of VAFs or *E. coli* pathotypes was compared between healthy and diarrheic animals and was analyzed for time courses. Together, 106 papers with 25,982 *E. coli* isolates from 27 countries tested for VAFs were included. F5, F17, and F41 fimbriae and heat-stable enterotoxin (ST) – VAFs of enterotoxigenic *E. coli* (ETEC) were significantly associated with calf diarrhea. On the contrary, ETEC VAF F4 fimbriae and heat-labile enterotoxin as well as enteropathogenic (EPEC), Shiga toxin-producing (STEC), and enterohemorrhagic *E. coli* (EHEC) were not associated with diarrhea. The prevalence increased overtime for ST-positive isolates, but decreased for F5- and STEC-positive isolates. Our study provides useful information about the history of scientific investigations performed in this domain so far, and helps to define etiological agents of calf disease, and to evaluate calves as reservoir hosts for human pathogenic *E. coli*.

## Introduction

*Escherichia coli* is one of the most common causes of diarrhea and septicemia in calves, affecting dairy, and beef production. Testing for *E. coli* is part of the routine epidemiological examination for diagnosis of calf diseases, alongside testing for rota- and coronavirus, *Cryptosporidium* spp., and *Salmonella* spp. Nevertheless, diarrhea in calves remains a major cause of annual financial losses for farmers. Moreover, as a very important reservoir of human pathogenic *E. coli*, calves can transmit this pathogen to humans (Cobbold et al., 2007).

Since the end of the nineteenth century, the role of *E. coli* as a pathogen of calves has been of interest to the scientific community worldwide. Reports concerning calf colibacteriosis are present in the works of Jensen (1903), Titze and Weichel (1908), Christiansen (1917); Carpenter and Woods (1924), and Smith and Orcutt (1925). The first scientific studies focused on calf diarrhea of very young calves (up to the first 4 days), which is called calf scours (in German: *Kälberruhr*, in French: *diarhée des veaux*). The general outcome of these studies was that “*Bacillus coli*” is a main etiological factor of calf diarrhea. They divided the disease into enteritis with and without septicemia. Additionally, a dose-dependent protective role was described for the colostrum. Colostrum-deprived calves or calves with delayed colostrum ingestion died after 1–3 days and large amounts of *E. coli* were isolated from their small and large intestines and other internal organs. Calves fed with an insufficient protective dose of colostrum developed various forms of bacterial infections, such as arthritis and omphalitis, alone or together with diarrhea. On the other hand, calves receiving a sufficient dose of colostrum had the highest odds of staying healthy. The main characteristics of *E. coli* isolated from diarrheic calves were: higher counts of bacteria from different parts of the intestine, and isolation of bacteria from internal organs or pathologically changed parts of the body (e.g., joints) as compared to healthy controls. These changes were proposed to stem from toxins released by bacteria that eventually altered the intestinal physiology. These assumptions were confirmed partially and extended in the later studies of Smith (1962). However, he disputed the role of *E. coli* as a causative agent of the disease in colostrum-fed calves, because of the failures encountered in establishing experimental infection and development of the disease in them. His later work, in which he developed a test for toxin production with calf ligated intestinal loops and oral inoculation of calves, helped to elucidate this issue (Smith and Halls, 1967). Using this model, Smith was able to induce fluid accumulation in intestinal loops and diarrhea in 6–20 h old infected calves.

The development of new laboratory tools for the analysis of pathogenic *E. coli* allowed for the verification of hypotheses raised by pioneers in this field. The main goal was to find a pattern for grouping isolates and to use this knowledge in disease diagnosis and prevention. Many approaches grouped bacteria by their reactivity with sera raised against standard strains. One of the first attempts to O-serogroup isolates from calf diarrhea was done by Bokhari and Ørskov (1952). Later, the antigen K99 as virulence-associated factor (VAF) associated with the pathogenesis of neonatal calf diarrhea was defined by Orskov et al. (1975) and Guinée et al. (1976). Also, biochemical features of isolates were investigated extensively, but without any remarkable success to specifically identify pathogenic *E. coli*. The emergence of DNA- and protein-based assays for the detection of VAF opened the door for much faster and more accurate evaluations of *E. coli* isolates (Grunstein and Hogness, 1975; Orskov et al., 1977; Moseley et al., 1980; Burnette, 1981; Espy et al., 2006).

The progress of science had a great impact on analytical typing methods for *E. coli*. It is now well accepted, that due to their high genotypic and phenotypic variation, *E. coli* can be subgrouped into many pathotypes. Commensalic *E. coli* is a common part of the intestinal microbiota of most mammals and birds. Only a small fraction of the *E. coli* population belongs to pathovars and pathotyping is based on the occurrence of VAF and virulence mechanisms (intestinal pathogenic *E. coli*) (Croxen et al., 2013) or by occurrence of *E. coli* in organs and tissues which are sterile in healthy hosts (extraintestinal pathogenic *E. coli*, e.g., uropathogenic *E. coli*). There are six major diarrheagenic *E. coli* pathotypes (Croxen et al., 2013). Enterotoxigenic *E. coli* (ETEC) is the confirmed main causative agent of neonatal calf diarrhea. Enteropathogenic *E. coli* (EPEC), Shiga toxin-producing *E. coli* (STEC including enterohemorrhagic *E. coli*/EHEC) are also often isolated from diarrheic and healthy calves, but their role in calf disease remains controversial. However, EPEC, STEC, and EHEC are important human pathogens for which cattle constitute a major reservoir. Enteroaggregative *E. coli* (EAEC), diffusely adherent *E. coli* (DAEC), and enteroinvasive *E. coli* (EIEC; including Shigella) are less known in cattle.

ETEC is characterized by the presence of specific adhesins and toxins. Adhesins involved in farm animal infection are F4, F5, F6, F17, and F41 fimbriae, all encoded by fimbrial operons (Nagy and Fekete, 1999, 2005). Relevant toxins are divided into two groups: heat-stable enterotoxin I and II (STI and STII) and heat-labile enterotoxin I and II (LTI and LTII) (Fleckenstein et al., 2010).

EPEC as well as enterohemorrhagic *E. coli* (EHEC) form attaching and effacing (A/E) lesions (Lai et al., 2013). This multistage process begins with initial attachment to the epithelial cell with surface associated filaments (EspA filaments) and bundle forming pili (Bfp). In a next step these bacteria use a type three secretion system to deliver several effector proteins including translocated intimin receptor (TIR) into the host cell followed by intimate attachment, mediated via intimin (*eaeA*)-TIR binding. Actin rearrangements then result in the formation of pedestal structures of A/E lesions (Clarke et al., 2003).

Shiga toxin-producing *E. coli* were first described as a pathotype 37 years ago (Konowalchuk et al., 1977). These bacterial strains are considered to be STEC if they are producing at least one of the Shiga toxins Stx1 or Stx2. The role of these bacteria as calf pathogens has not been conclusively elucidated. There have been cases of fatal STEC infections in cattle, despite their lack of expression of globotriaosylceramide (Gb3), a receptor for Stx cellular internalization, on their vascular endothelium (Pruimboom-Brees et al., 2000). Recent studies show that receptor binding is different between Stx1 and Stx2 and might involve more than one glycan (Gallegos et al., 2012). STEC that are able to induce A/E lesions are grouped as enterohemorrhagic *E. coli* (EHEC). Cattles are frequent shedders of EHEC with approximately 75% of human disease outbreaks linked to bovine derived products or cattle (Nguyen and Sperandio, 2012).

EAEC was initially recognized by a specific adhesion pattern on HEp-2 cells forming cobble-stone-like aggregates (Weintraub, 2007). EAEC adherence is mediated by aggregative adherence fimbriae (AAF) coded on a virulence pAA plasmid. In addition, EAEC form mucoid biofilms and secret cytotoxins (e.g., plasmid-encoded toxin Pet) that are toxic to epithelial cells. The enterotoxins “enteroaggregative *E. coli* ST (EAST1)” and “*Shigella* enterotoxin 1 (ShET1)” were associated with EAEC, but they can be found also in other pathotypes (Croxen and Finlay, 2010; Ruan et al., 2012).

DAEC are defined by their diffuse adherence (DA) pattern on HEp-2 or HeLa cells (Servin, 2005). DAEC express fimbriae like Dr and F1845 and/or afimbrial adhesins (Afa) which are responsible for adhesion to the epithelium and which are considered as the main VAFs for this pathotype. Adhesion results in an effacement of microvilli and a disruption of enzymes involved in intestinal secretion, which contribute to diarrhea.

NTEC produce several powerful toxins. Originally, NTEC were mainly characterized by the secretion of cytotoxic necrotizing factors (CNF 1, 2, 3) (Orden et al., 2007). Additionally they can produce toxins like hemolysin and cytolethal distending toxin (cdt). NTEC were associated with intestinal and extraintestinal infections in both humans and animals (Kaper et al., 2004).

Four types of hemolysin have been identified in *E. coli* (Lorenz et al., 2013). Alpha-hemolysin (hlyA) is produced by many strains associated with urinary tract infections. Phage-carried enterohemolysin (ehxA) is frequently associated with the STEC pathotype. The role of the bacteriophage carried enterohemolysin (e-hlyA) is so far not known. Presence of the silent hemolysin (sheA) has been confirmed in most of the *E. coli* pathotypes. Alpha-hemolytic *E. coli* were frequently isolated from healthy as well as diseased animals and humans and their specific physiological or pathophysiological role remains unclear (Schierack et al., 2006, 2011).

Common diagnostic methods for pathotyping *E. coli* rely on the detection of genes/gene products and/or proteins which focus on toxins and adhesion factors. Biological assays are occasionally added to investigate and test for virulence mechanisms or to quantify biological activities of VAFs. One popular method for the prediction of pathogenicity is typing of somatic (O) and flagellar (H) antigens. Actually, there are 181 O and 53 H antigens (Lacher et al., 2014). Several O groups are more prevalent in single pathotypes (Ewers et al., 2014). Other tools for the characterization of isolates are various genotyping methods e.g., pulsed field gel electrophoresis (PFGE), random amplified polymorphic DNA (RAPD), multilocus sequence typing (MLST), multiple loci VNTR analysis (MLVA), and the Clermont method (Clermont et al., 2000; Foley et al., 2004).

Over the last decades numerous studies were published about *E. coli* in calves describing single events in circumscribed areas with controversial results. However, systematic reviews and even meta-analyses of previous studies are still rare endeavors, reflecting a trend in the field of veterinary medicine. Such meta-analyses are strong tools to clarify the role of bacteria in disease of a specific host group, the role of a host species as reservoir for other species and epidemiological trends which can help in the evaluation of disease defense strategies (e.g., vaccination), optimization of existing diagnostic protocols and indication of new research topics. Our meta-analysis reviews all published and available data between 1951 and 2013 concerning the presence of VAFs in *E. coli* isolated from diarrheic and healthy calves and discusses the role of *E. coli* pathotypes in disease, the role of calves as pathogen reservoir and the epidemiological trends in this field over the past decades.

## Materials and methods

### Literature search and eligibility criteria

**Figure 1:**
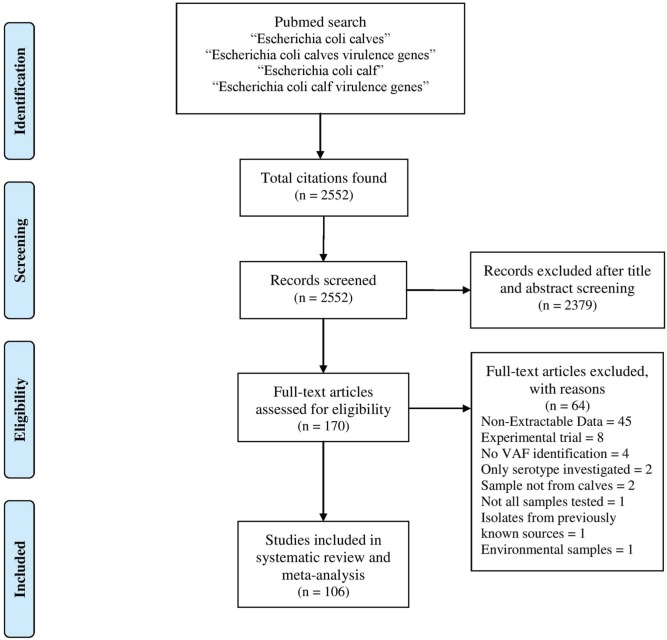
**Flow diagram for the selection of manuscripts**.

The PubMed database was searched for studies published from 1st of January 1951 to 31st of December 2013 with the following phrases: “*E. coli* calves,” “*E. coli* calves virulence genes,” “*E. coli* calf,” and “*E. coli* calf virulence genes.” Manual revision was conducted on all displayed publications and first selections were based on information in the titles and/or abstracts. Selected publications had to be available for downloading and had to contain extractable data in English about the presence of VAF in *E. coli* isolated from calves. Selection of studies and extraction of data was done independently by the authors RK and MB and then compared. All discrepancies were reviewed by the third author PS. The study selection workflow is presented in Figure 1.

### Data extraction

Data was extracted by RK and MB. The compiled information contains: year(s) and country of sampling, number of animals sampled, presence of diarrhea, number of isolates analyzed, number of isolates positive for VAFs, serotypes, diagnostic methods, and biological assays. Isolates were divided into four groups depending on the animals' health status: diarrheic, healthy, mixed (studies did not distinguish between diarrheic and healthy), unknown. Collected raw data was stored in a relational database created specifically for this study.

### Data preprocessing

Due to the non-homogeneous nomenclature the naming convention of VAFs was unified. For example, isolates originally annotated as *eae*, *eaeA*, and/or intimin were re-coded as *eaeA*-positive. Another reason for unification was the diversity in analyzed genes. In order to improve data processing we aggregated subtypes of genes as one gene (e.g., all Stx2e and Stx2 are grouped as Stx2). These two aforementioned modifications are shown in Supplementary Table 1. Whenever isolates were collected over more than 1 year, a consensus year was the average between the year of beginning and end of the gathering of isolates.

### Missing data imputation

Forty-one (39%) publications did not contain precise information about the time frame in which isolates were collected. The probable year of collection for such a study was approximated by the linear model y = a ^*^ x fitted to data from all publications with clearly indicated collection periods. The isolation year was treated as the response variable and the publication year was used as the explanatory variable. This approximation resulted in the factor of 0.9977: isolation_year = publication_year ∗ 0.9977 (*p*-value: <2.2e-16, adjusted *R*^2^ = 1).

### Pathotyping

We analyzed data as follows: (1) Data for all VAFs were analyzed regarding the prevalence of single VAFs with no mentioned affiliation to VAF patterns (e.g., 54% of all isolates were *eaeA*-positive, 37% of all isolates were Stx-positive, but with no information about the prevalence of EHEC = Stx + *eaeA*). (2) For EPEC, STEC, and EHEC we analyzed and presented the prevalence of single VAFs and affiliation of all isolates to possible VAF patterns (e.g., one isolate was *eaeA*-positive = EPEC, one isolate was Stx-positive = STEC, one isolate was *eaeA*- and Stx-positive = EHEC). This was possible due to a large number of relevant publications.

### Data processing and statistical analysis

The prevalence of VAFs or *E. coli* pathotypes was compared between healthy and diarrheic animals using the chi-square test of independence with Yates's continuity correction. The same statistical test was used to determine relationships between the prevalence of ETEC, EPEC, STEC, EHEC, and the animal health status. Correlations between the prevalence of a VAF and the year of isolation of *E. coli* was determined with the Pearson correlation coefficient on normally distributed data. The false discovery rate of all statistical tests was controlled by applying Benjamini and Hochberg correction to all obtained *p*-values (Benjamini and Hochberg, 1995). Confidence intervals of the prevalence for each VAF were estimated using the Wilson score interval with continuity correction. This method was chosen because of its good coverage properties (Brown et al., 2001).

This study was conducted following the guidelines for reporting meta-analysis of observational studies in epidemiology (MOOSE) (Stroup et al., 2000) and preferred reporting items for systematic reviews and meta-analyses (PRISMA) statement (Moher et al., 2009) with PRISMA 2009 Checklist (Supplementary File 1).

All calculations were performed using the R software (R Development Core Team, 2013). All figures were made using the ggplot2 package (Wickham, 2010) implemented in the R programming environment.

## Results

Our systematic review and meta-analysis includes data from 106 studies from 2552 citations identified in the Pubmed database (Supplementary File 2). These studies represent data about bacteria isolated in 27 countries (Supplementary Figure 1) and published in 37 journals (Supplementary Figure 2). The results of our search are summarized in Supplementary Table 2. In all, we gathered information from 25,982 isolates tested for VAFs.

### Methods for characterization of isolates

The methods used for VAF detection in *E. coli* are summarized in Supplementary Table 3. PCR was used most often for VAF detection. More than a half of papers contained information about serotypes. Clonal relationship assays and antimicrobial susceptibility tests were analyzed in nearly one-fifth of all publications under review.

### VAF: overall prevalence and association with health status

The database covers information about 61 VAFs (genes, operons, and/or expressed antigens). Each publication presented information about 1–27 VAFs (median = 3.5 VAFs per publication). Only the most prevalent and pathotype-associated VAFs were chosen for further analysis and discussion. However, all other VAFs have also been listed in Table 1 and Supplementary Table 4. In addition, the relation between particular VAFs and an animal's health status is shown in Table 1. In summary 16 VAFs were associated with animals' health status with *p*-value lower than 0.05 (lowest *p* in these group 10^−52^). Identification of five VAFs was associated with healthy calves, while 11 of them with diseased.

**Table 1:** **Comparison of study groups (healthy and diarrheic)**.

**Group**	**Diarrheic**				**Healthy**				***P*-value**
**VAF name**	**Number of publications**	**Isolates positive**	**Isolates tested**	**Proportion of positives**	**Number of publications**	**Isolates positive**	**Isolates tested**	**Proportion of positives**	
aaf	3	0	155	0.0000	1	0	56	0.0000	NA
Aerobactin	5	114	174	0.6552	1	49	56	0.8750	0.0085
Afa	7	423	1384	0.3056	2	1	79	0.0127	2.356e-07
bfp	6	3	353	0.0085	3	0	117	0.0000	0.9756
cdt	6	78	1271	0.0614	2	7	77	0.0909	0.6468
cif	1	15	255	0.0588	1	0	21	0.0000	0.7666
CNF	10	135	1880	0.0718	6	55	1356	0.0406	9.089e-04
CS31	7	183	646	0.2833	2	1	145	0.0069	1.404e-11
eaeA	33	1203	9779	0.1230	29	856	4295	0.1993	7.766e-31
EAF	3	3	160	0.0187	2	0	61	0.0000	0.934
EAST	5	45	148	0.3041	1	18	56	0.3214	1
EFA1	1	15	16	0.9375	1	23	23	1.0000	0.9756
eibG	1	0	16	0.0000	1	0	23	0.0000	NA
ent	1	0	61	0.0000	1	0	56	0.0000	NA
escV	1	14	61	0.2295	1	16	56	0.2857	0.9005
F17	15	735	2328	0.3157	4	231	854	0.2705	0.0437
F1845	1	0	14	0.0000	1	3	122	0.0246	1
F41	15	293	2030	0.1443	6	6	991	0.0061	2.521e-31
F5	36	719	5566	0.1292	12	42	1855	0.0226	1.580e-37
fim	1	60	61	0.9836	1	56	56	1.0000	1
fyuA	2	36	76	0.4737	1	29	56	0.5179	0.9756
hcp	1	3	16	0.1875	1	0	23	0.0000	0.2138
hly	25	455	1661	0.2739	15	283	1368	0.2069	9.274e-05
ibe	1	45	61	0.7377	1	39	56	0.6964	0.9756
iha	2	21	58	0.3621	1	23	23	1.0000	3.281e-06
inv	2	0	75	0.0000	1	0	56	0.0000	NA
ipa	3	0	107	0.0000	1	0	56	0.0000	NA
kpsMII	2	4	103	0.0388	1	1	56	0.0179	0.9756
ldaE	1	7	16	0.4375	1	0	23	0.0000	0.0069
LT	5	3	1141	0.0026	1	0	56	0.0000	1
LTI	7	0	409	0.0000	3	0	806	0.0000	NA
LTII	3	4	146	0.0274	1	0	20	0.0000	1
malX	2	3	103	0.0291	1	5	56	0.0893	0.3334
paa	1	15	16	0.9375	1	23	23	1.0000	0.9756
Pap	7	299	1248	0.2396	1	42	112	0.3750	0.0071
pic	2	0	77	0.0000	1	0	56	0.0000	NA
saa	4	119	248	0.4798	3	60	863	0.0695	9.715e-52
sfa	4	37	998	0.0371	1	3	56	0.0536	0.9756
STI	17	172	2178	0.0790	8	7	1261	0.0056	1.531e-19
STII	7	50	1043	0.0479	3	0	806	0.0000	3.924e-09
stx	4	27	94	0.2872	2	20	130	0.1538	0.0584
stx1	34	745	3562	0.2092	31	677	3577	0.1893	0.0877
stx2	34	402	3562	0.1129	31	654	3577	0.1828	7.275e-16
toxB	1	11	16	0.6875	1	22	23	0.9565	0.1343
traT	1	58	61	0.9508	1	54	56	0.9643	1
uidA	1	59	61	0.9672	1	51	56	0.9107	0.5763
etpD	NA	NA	NA	NA	1	2	13	0.1538	NA
aap	1	0	16	0.0000	NA	NA	NA	NA	NA
esp	3	32	212	0.1509	NA	NA	NA	NA	NA
F18	1	0	14	0.0000	NA	NA	NA	NA	NA
F4	3	0	716	0.0000	NA	NA	NA	NA	NA
F6	1	82	666	0.1231	NA	NA	NA	NA	NA
HPI	1	27	42	0.6429	NA	NA	NA	NA	NA
iroN	1	4	42	0.0952	NA	NA	NA	NA	NA
modD	1	4	42	0.0952	NA	NA	NA	NA	NA
shf	1	3	16	0.1875	NA	NA	NA	NA	NA

NA, not applicable; P-value, value for difference between healthy and diarrheic.

### Enterotoxigenic *Escherichia coli*

The presence of ETEC VAFs was investigated in 9597 isolates from 27 countries in 51 publications. ST and LT were present in 4.3% (254/5849 isolates) and 0.3% (7/2672) isolates, respectively (Table 2).

**Table 2:** **Data about ETEC VAFs**.

**Group name**	**Number of publications**	**Isolates positive**	**Isolates tested**	**Percent positive**
F4	4	0	810	0.0
F5	44	795	8346	9.5
F6	2	82	760	10.8
F17	17	966	3182	30.4
F41	19	391	3515	11.1
LT	6	3	1291	0.2
LTI	9	0	1215	0.0
LTII	3	4	166	2.4
LT overall	15	7	2672	0.3
STI	22	192	3906	4.9
STII	10	62	1943	3.2
ST overall	22	254	5849	4.3

ST was found in 7% of isolates from diseased and in 0.3% from healthy animals. STI (4.9%) was isolated more often than STII (3.2%). LT was almost absent in isolates from both diarrheic and healthy calves. EAST was found with similar prevalence in isolates from healthy (32.1%) and diarrheic calves (30.4%, Table 1). The prevalence of various ETEC adhesins is shown in Table 2. F5, F17, and F41 fimbriae were the most frequently studied. They were isolated from 9.5, 30.4, and 11.1% animals, respectively. All of them were found more often in diarrheic, than in healthy animals (Table 1). F5, F17, and F41 fimbriae were isolated 5.7, 1.2, and 23.7 times more frequently from diarrheic calves than from healthy ones. F4 and F6 were tested for only in four and two publications, respectively. F4 was not found in analyzed samples (810 isolates tested). F6 fimbriae were detected in 12.3% (82 of 666) of diarrheic isolates, but no data about prevalence in healthy animals was available.

**Figure 2:**
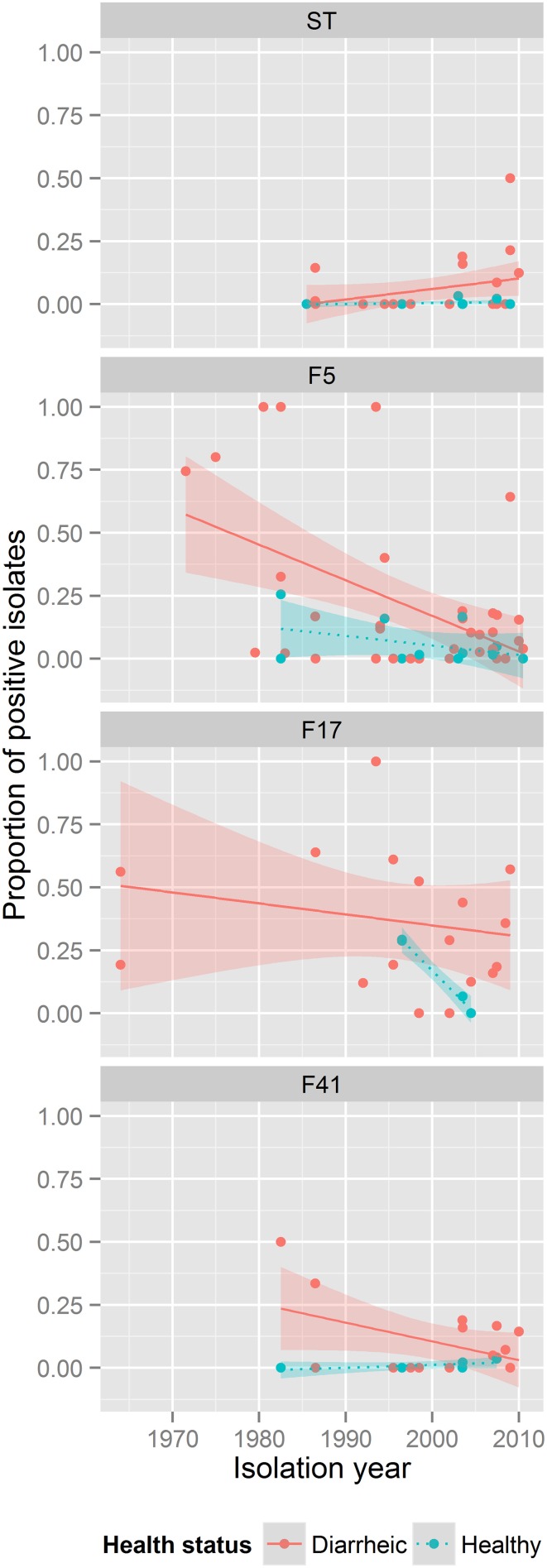
**ETEC VAF prevalence in between 1962 and 2013**. Each subpanel corresponds to one VAF. The color of dots describes health status of animals: red, diarrheic animals; blue, healthy animals. The lines show general tendencies by approximating the relationship between the year of isolation and the prevalence of a VAF using a linear model y = ax + b. The shaded areas around lines represent confidence intervals.

The prevalence of ST-positive isolates in diarrheic and healthy calves increased over time (*p* < 0.05). F5 prevalence decreased over time in diseased and healthy animals (*p* < 0.0005), while prevalence did not change for the other VAFs within this time (Figure 2; Supplementary Figure 3).

### Enteropathogenic *Escherichia coli*

The presence of EPEC VAFs (one *eaeA* gene + absence of Shiga toxins or Shiga toxin genes) was investigated in 12,246 isolates from 18 countries in 37 publications.

EPECs were found more often in healthy than in diarrheic animals with 7.5% of isolates from diarrheic animals (709 from 9448 isolates) and 14.6% of isolates from healthy animals (383 from 2629 isolates) being EPEC (Table 3).

**Table 3:** **Data about EPEC, STEC, EHEC**.

**Pathotype**	**EPEC**			**STEC**			**EHEC**		
**Group**	**Isolates tested**	**Isolates positive**	**Number of publications**	**Isolates tested**	**Isolates positive**	**Number of publications**	**Isolates tested**	**Isolates positive**	**Number of publications**
Diarrheic	9448	709	27	3248	592	36	9014	545	31
Healthy	2629	383	19	3147	610	28	4120	442	27
Mixed	0	0	0	130	66	1	130	64	1
Unknown	169	0	3	1528	386	10	562	146	5
Total	12246	1092	37	8053	1654	61	13826	1197	50

### Shiga toxin-producing *Escherichia coli*

**Figure 3:**
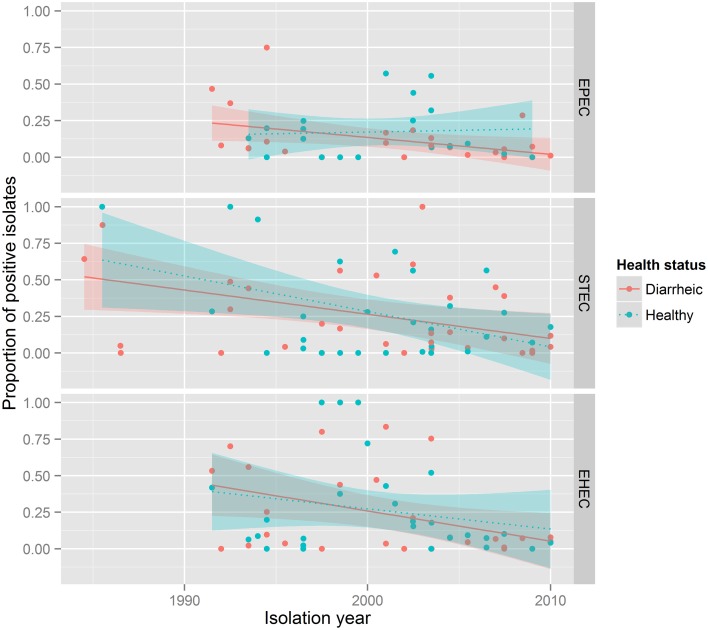
**STEC, EPEC, EHEC prevalence in between 1986 and 2010**. Each subpanel corresponds to one pathotype. The color of dots describes health status of animals: red, diarrheic animals; blue, healthy animals. The lines show general tendencies by approximating the relationship between the year of isolation and the prevalence of a pathotype using a linear model y = ax + b. The shaded areas around lines represent confidence intervals.

**Figure 4:**
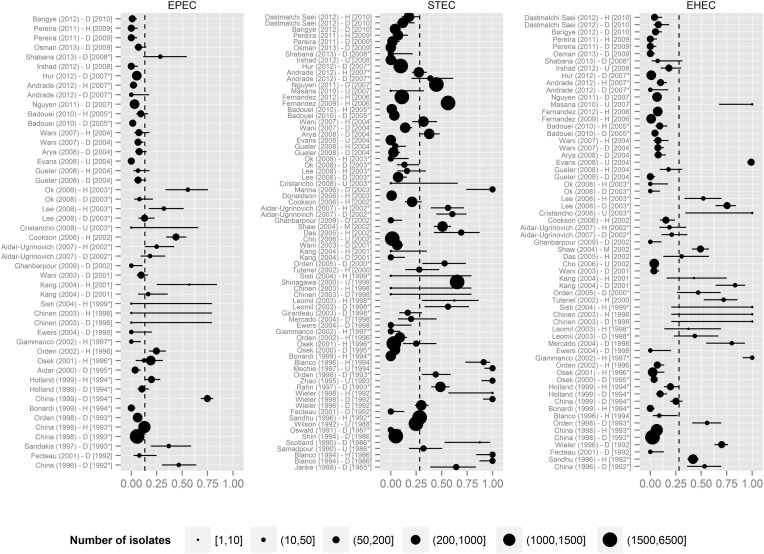
**Prevalence of STEC, EPEC, EHEC in the manuscripts under review**. The dashed vertical lines represent the mean prevalence of the given pathotype of all studies. Labels contain information about the first author of the study, the year of publication, the health status of animals (H, healthy; D, diarrheic; M, mixed; U, unknown) and the year of isolation (with the asterisk if isolation date was extrapolated). Horizontal lines represent the 0.95 confidence intervals. Point size represents number of isolates for a pathotype. Data were sorted according to the year of isolation.

The presence of STEC VAFs was investigated in 8053 isolates from 19 countries in 61 publications (Table 3). The average prevalence of STECs isolated from healthy animals was 19.4% and from diseased animals 18.2%. Only 51 publications including 5676 isolates determined co-occurrence of toxins: 344 isolates were Stx1-positive (6.1%), 405 isolates were Stx2+ (7.1%), and in 259 isolates both Shiga toxin genes were present (4.6%). STEC was not associated with diarrhea. STEC prevalence decreased over time (*p*-value < 0.001, Figure 3). Interestingly, STEC prevalence is remarkably smaller in contemporary than in older studies (Figure 4).

### Enterohemorrhagic *Escherichia coli*

The presence of EHEC VAFs (one *eaeA* gene + Shiga toxins or Shiga toxin genes) was investigated in 13,826 isolates from 18 countries in 50 publications. The prevalence of EHEC independent of health status was 8.7% (1197 isolates were EHEC) with 6% in diarrheic and 10.7% in healthy calves (*p* < 10^−15^, Table 3). Forty four papers including 12,378 isolates differentiated between Stx1 and Stx2: 659 isolates were Stx1 + *eae*A-positive, 180 isolates were Stx2 + *eaeA*-positive and 118 isolates were Stx1 + Stx2 + *eaeA*-positive. EHEC prevalence did not decreased over time (Figures 3, 4).

### Other VAFs

Afa and F1845 are typical for DAEC. The presence of DAEC VAFs was investigated in 1568 isolates from 10 countries in nine publications. Afa was found significantly more often in isolates from diarrheic (30.6% of isolates) as compared to isolates from healthy animals (1.3%; *p* < 10^−6^). F1845 was investigated only in two publications and three from 136 isolates (2.2%) were positive (Table 1). Aggregative adhesion fimbria (aaf) expression in EAEC was not found in any of 147 isolates tested in three publications from three countries.

The presence of the cytotoxic necrotizing factor (CNF) was investigated in 2125 isolates from nine countries in 14 publications. CNF was reported 1.8 times more often in isolates from diarrheic (7.2%) than in isolates from healthy animals (4.1%; *p* < 0.001). *E. coli* hemolysins (hly, including all four types) were more often reported in isolates from diarrheic animals (27.4%) than in isolates from healthy animals (20.7%; *p* < 10^−4^).

### Biological assays

Phenotypic characterization can improve pathotyping of *E. coli*. *In vitro* cell-based or *ex vivo* assays are the primary choice for this purpose. In Supplementary Table 5 we summarize such biological assays. The most often used was the Vero cytotoxicity assay, which is found in 27 manuscripts. Cells or cell lines were rarely used to investigate adhesion/adhesion pattern of *E. coli*. Enterotoxin production was done in 10 publications for which ligated intestinal loops or mouse infant assays were used.

## Discussion

Systematic epidemiological reviews and meta-analyses on publications of *E. coli* as calf pathogen and the role of calves as human pathogenic *E. coli* reservoir have not been carried out so far. Therefore, the present study was undertaken to summarize data from over 60 years of research in this field. We included all eligible studies from the Pubmed database. Data were extracted and stored in a relational database created specifically for this research and analyzed with R software (R Development Core Team, 2013).

ETECs are regarded as major agents in the etiology of calf diarrhea. One of the first steps of ETEC virulence mechanisms is adhesion to the intestinal epithelium, which is mediated by fimbrial adhesins, mainly the F4, F5, F6, F17, and F41 fimbriae. According to our analysis the presence of F4 is not associated with diarrhea. F4 fimbriae-positive strains were first reported in pigs and are the most common VAF in porcine ETEC. Therefore, absence of F4 in calf isolates is not surprising. The role of F6 fimbriae in the etiology of calf diarrhea remains unclear, owing to the lack of information about healthy animals. F17 have been implicated in the pathogenesis of calf diarrhea and we show that F17 is found more often in diarrheic than in healthy calves (*p* < 0.05); yet, there is high prevalence of F17 positive isolates in healthy animals (27%). We propose two explanations: Firstly, the F17 fimbrium requires the presence of other VAFs to participate in the etiology of diarrhea. Secondly, F17 fimbriae genes are present in *E. coli*, as detected by the most common method PCR, but are not functionally expressed. Unfortunately, resolving this issue requires more in-depth research.

F5 and F41 fimbriae were highly associated with the presence of diarrhea (*p* < 10^−36^ and *p* < 10^−30^). F41 prevalence has the highest diarrheic to healthy ratio, which justifies a major role of this pilus in ETEC pathogenesis. F5 fimbriae were the first VAF identified in ETEC from diarrheic isolates (Guinée et al., 1976). They are also the most often investigated VAF in our ETEC meta-analysis. This gave us the opportunity to show changes in prevalence of F5 over time. A significant drop in F5 prevalence over the past years was observed. This outcome can be explained by the commonness of vaccination against this ETEC antigen (Moon and Bunn, 1993; Crouch et al., 2001), which could have led to negative selection against F5 fimbriae-positive bacteria coincident with success in disease control.

After attachment to the intestinal mucosa, ETEC initiate their pathogenic actions by secretion of toxin(s). It has been proposed, that ST plays a major role in the pathogenesis of calf diarrhea (Nagy and Fekete, 1999). Indeed, ST toxin was detected frequently only in diarrheic isolates, while LT was reported only in seven of 2672 isolates. The role of EAST toxin in the pathogenesis of calf diarrhea seems to be doubtful. Although the overall prevalence of EAST was high, it was represented in diarrheic and healthy samples in similar frequencies. Recently, published data by Ruan et al. (2012) indicated that an EAST1-positive isolate alone is not able to cause diarrhea in a 5-day old gnotobiotic pig model, and suggests that EAST1 is not likely a VAF in ETEC-associated diarrhea. Additionally, EAST1 was detected with high prevalence in clinically healthy individuals of other domestic and wild animal species (Römer et al., 2012; Frömmel et al., 2013) which also puts to doubt whether EAST1 has a prominent role in diarrhea.

The readers of this manuscript should raise an important question at this moment. If the highest prevalence of VAF from *E. coli* isolated from diarrhea is between 5 and 15%, what is happening with the rest of isolates? Are those isolates commensalic *E. coli* or are there still many unidentified adhesins and toxins which may contribute to pathogenesis? *E. coli* can be isolated from each diarrheic sample but might not be the adequate causative agent of disease. A favorable differential diagnosis of infectious agents of calf diarrhea consists of testing for rota- and coronavirus, *Salmonella* spp., and *Cryptosporidium* spp.. Unfortunately, information about such investigations was sparse in the context of our meta-analysis. Only 22 publications mentioned additional testing for at least one of the above mentioned pathogens. Other important causes of diarrhea are non-infectious, and related to housing, management, and feeding routines (Ortiz-Pelaez et al., 2008). Isolation of pathogenic *E. coli* from most of these cases should be rare and are not associated with disease. The inclusion of a questionnaire into relevant studies could help in obtaining valuable information on how the environment influences animal health, but such additional data were mentioned only in 15 publications.

The next interesting topic raised in our work is the prevalence of STEC, EPEC, and EHEC in calves. The high prevalence of STEC, EPEC, and EHEC in healthy and diarrheic animals shows, that these animals can be considered as reservoirs for human pathogenic *E. coli*. The role of STEC, EPEC, and EHEC as causative agents of diarrhea in calves have been widely investigated (Dean-Nystrom et al., 1998; Menge et al., 1999; Pruimboom-Brees et al., 2000; Hoey et al., 2002). Lack of Stx receptor in vascular endothelium makes cattle a perfect candidate as a reservoir of STEC/EHEC as it is not possible for them to develop systemic disease. Other actions of STEC/EHEC like immunomodulation (Menge et al., 1999) and intestinal colonization (Etcheverría and Padola, 2013) help them to survive and propagate in the host intestine. Major questions remain to be answered as to whether these processes contribute to pathogenesis of diarrhea. Our comparison of the prevalence of STEC, EPEC, and EHEC in diarrheic and healthy calves showed that none of these pathotypes is associated with disease, and these pathotypes were more often detected in healthy animals. But can cattle have any profit from the presence of these bacteria? An interesting hypothesis was raised in the works of Ferens and Hovde (2000), Basu et al. (2003) and Ferens et al. (2004, 2006), that Stx production can mitigate bovine leukemia virus (BLV)-induced disease in cattle. This hypothesis could explain the strong species-pathotype association, but to our knowledge no survey study about BLV-STEC/EHEC prevalence correlation has been conducted so far.

Another intriguing observation is the decrease in the prevalence of STEC over time as shown in Figures 3, 4. We assumed that prevalence might be influenced by the diagnostic methods used. To verify this, we created additional forest plots with information about methods used in the study (Supplementary Figure 4). The analysis of this results shows, that our assumption was wrong.

DAEC and EAEC are well-known as a diarrhea causing agents in humans (Servin, 2005; Weintraub, 2007), but there is scant information about their prevalence in cattle and calves. In the case of DAEC, data from nine studies show that the prevalence of this pathotype is similar to the prevalence of ETEC VAFs. Therefore, it seems that cattle might be affected by DAEC. Attention should be paid on EAEC and DAEC in next years.

Analysis of the methods used in studies appraised in this work is worth a small separate discussion. Supplementary Figure 5 shows high impact of DNA-based methods on pathotyping. As these tools are fast and reliable, an additional confirmation for pathogenic phenotypes using phenotyping assays seems to be possible and useful. However, such assays were rarely applied. Newly developed technologies seem to be an answer for the need of quick, reliable and high-throughput functional analysis of isolated *E. coli* (Han and Lee, 2006; Sorek and Cossart, 2010; Frömmel et al., 2013; Rödiger et al., 2013).

It is our duty to mention the drawbacks of our work. Due to the lack of reports about several VAFs (e.g., F6), performance of planned analyses was not possible. Unfortunately in many studies different sets of genes and health status of animals were investigated, therefore presented analyses for each VAF are composed of sets of various number of isolates. The atypical structure of data prohibited fitting with more advanced statistical models used often in meta-analysis. One of our goals was to evaluate the spatial distribution of VAFs and pathotypes, but it was not feasible due to the geographical sparseness of the data. Still, we think that our systematic review and meta-analysis is a good way to summarize epidemiological status of pathogenic *E. coli* in calves. Our study provides useful information about the history of scientific investigations performed so far and helps understand their influence on forthcoming studies. Our data suggest new trends for future work concerning *E. coli* in calves as an etiological agent of disease or in carrier state. We hope that our study will highlight the need to develop a unified framework for future research on pathogenic *E. coli* of calves.

### Conflict of interest statement

The authors declare that the research was conducted in the absence of any commercial or financial relationships that could be construed as a potential conflict of interest.

## References

[B1] Basu I. Ferens W. A. Stone D. M. Hovde C. J. (2003). Antiviral activity of shiga toxin requires enzymatic activity and is associated with increased permeability of the target cells. Infect. Immun. 71, 327–334. 10.1128/IAI.71.1.327-334.2003 12496182 PMC143405

[B2] Benjamini Y. Hochberg Y. (1995). Controlling the false discovery rate: a practical and powerful approach to multiple testing. J. R. Stat. Soc. B (Methodol.) 57, 289–300.

[B3] Bokhari S. M. H. Ørskov F. (1952). O-grouping of *E. coli* strains isolated from cases of white scours. Acta Pathol. Microbiol. Scand. 30, 87–89. 10.1111/j.1699-0463.1952.tb00164.x 14933043

[B4] Brown L. D. Cai T. T. DasGupta A. (2001). Interval estimation for a binomial proportion. Stat. Sci. 16, 101–133 10.1214/ss/1009213286

[B5] Burnette W. N. (1981). “Western Blotting”: electrophoretic transfer of proteins from sodium dodecyl sulfate-polyacrylamide gels to unmodified nitrocellulose and radiographic detection with antibody and radioiodinated protein A. Anal. Biochem. 112, 195–203. 10.1016/0003-2697(81)90281-5 6266278

[B6] Carpenter C. Woods G. (1924). The distribution of the colon-aerogenes group of bacteria in the alimentary tract of calves. Cornell Vet. 14, 218–225.

[B7] Christiansen M. (1917). Bakterien der Typhus-coli-Gruppe im Darm von gesunden Spankkalbern und bei deren Darminfektion. Zbl. Bakt. 79, 196.

[B8] Clarke S. C. Haigh R. D. Freestone P. P. E. Williams P. H. (2003). Virulence of enteropathogenic *Escherichia coli*, a global pathogen. Clin. Microbiol. Rev. 16, 365–378. 10.1128/CMR.16.3.365-378.2003 12857773 PMC164217

[B9] Clermont O. Bonacorsi S. Bingen E. (2000). Rapid and simple determination of the *Escherichia coli* phylogenetic group. Appl. Environ. Microbiol. 66, 4555–4558. 10.1128/AEM.66.10.4555-4558.2000 11010916 PMC92342

[B10] Cobbold R. N. Hancock D. D. Rice D. H. Berg J. Stilborn R. Hovde C. J.. (2007). Rectoanal junction colonization of feedlot cattle by *Escherichia coli* O157:H7 and its association with supershedders and excretion dynamics. Appl. Environ. Microbiol. 73, 1563–1568. 10.1128/AEM.01742-06 17220263 PMC1828767

[B11] Crouch C. F. Oliver S. Francis M. J. (2001). Serological, colostral and milk responses of cows vaccinated with a single dose of a combined vaccine against rotavirus, coronavirus and *Escherichia coli* F5 (K99). Vet. Rec. 149, 105–108. 10.1136/vr.149.4.105 11504200

[B12] Croxen M. A. Finlay B. B. (2010). Molecular mechanisms of *Escherichia coli* pathogenicity. Nat. Rev. Microbiol. 8, 26–38. 10.1038/nrmicro2265 19966814

[B13] Croxen M. A. Law R. J. Scholz R. Keeney K. M. Wlodarska M. Finlay B. B. (2013). Recent advances in understanding enteric pathogenic *Escherichia coli*. Clin. Microbiol. Rev. 26, 822–880. 10.1128/CMR.00022-13 24092857 PMC3811233

[B14] Dean-Nystrom E. A. Bosworth B. T. Moon H. W. O'Brien A. D. (1998). *Escherichia coli* O157:H7 requires intimin for enteropathogenicity in calves. Infect. Immun. 66, 4560–4563. 9712821 10.1128/iai.66.9.4560-4563.1998 PMC108559

[B15] Espy M. J. Uhl J. R. Sloan L. M. Buckwalter S. P. Jones M. F. Vetter E. A.. (2006). Real-time PCR in clinical microbiology: applications for routine laboratory testing. Clin. Microbiol. Rev. 19, 165–256. 10.1128/CMR.19.1.165-256.2006 16418529 PMC1360278

[B16] Etcheverría A. I. Padola N. L. (2013). Shiga toxin-producing *Escherichia coli*: factors involved in virulence and cattle colonization. Virulence 4, 366–372. 10.4161/viru.24642 23624795 PMC3714128

[B17] Ewers C. Stamm I. Stolle I. Guenther S. Kopp P. A. Fruth A. Wieler L. H.. (2014). Detection of Shiga toxin- and extended-spectrum β-lactamase-producing *Escherichia coli* O145:NM and Ont:NM from calves with diarrhoea. J. Antimicrob. Chemother. 69, 2005–2007. 10.1093/jac/dku042 24595804

[B18] Ferens W. A. Cobbold R. Hovde C. J. (2006). Intestinal Shiga toxin-producing *Escherichia coli* bacteria mitigate bovine leukemia virus infection in experimentally infected sheep. Infect. Immun. 74, 2906–2916. 10.1128/IAI.74.5.2906-2916.2006 16622229 PMC1459712

[B19] Ferens W. A. Grauke L. J. Hovde C. J. (2004). Shiga toxin 1 targets bovine leukemia virus-expressing cells. Infect. Immun. 72, 1837–1840. 10.1128/IAI.72.3.1837-1840.2004 14977999 PMC356031

[B20] Ferens W. A. Hovde C. J. (2000). Antiviral activity of shiga toxin 1: suppression of bovine leukemia virus-related spontaneous lymphocyte proliferation. Infect. Immun. 68, 4462–4469. 10.1128/IAI.68.8.4462-4469.2000 10899843 PMC98349

[B21] Fleckenstein J. M. Hardwidge P. R. Munson G. P. Rasko D. A. Sommerfelt H. Steinsland H. (2010). Molecular mechanisms of enterotoxigenic *Escherichia coli* infection. Microbes Infect. 12, 89–98. 10.1016/j.micinf.2009.10.002 19883790 PMC10647112

[B22] Foley S. L. Simjee S. Meng J. White D. G. McDermott P. F. Zhao S. (2004). Evaluation of molecular typing methods for *Escherichia coli* O157:H7 isolates from cattle, food, and humans. J. Food Prot. 67, 651–657. 15083714 10.4315/0362-028x-67.4.651

[B23] Frömmel U. Böhm A. Nitschke J. Weinreich J. Groß J. Rödiger S.. (2013). Adhesion patterns of commensal and pathogenic *Escherichia coli* from humans and wild animals on human and porcine epithelial cell lines. Gut Pathog. 5:31. 10.1186/1757-4749-5-31 24188314 PMC4177131

[B24] Gallegos K. M. Conrady D. G. Karve S. S. Gunasekera T. S. Herr A. B. Weiss A. A. (2012). Shiga toxin binding to glycolipids and glycans. PLoS ONE 7:e30368. 10.1371/journal.pone.0030368 22348006 PMC3278406

[B25] Grunstein M. Hogness D. S. (1975). Colony hybridization: a method for the isolation of cloned DNAs that contain a specific gene. Proc. Natl. Acad. Sci. U.S.A. 72, 3961–3965. 10.1073/pnas.72.10.3961 1105573 PMC433117

[B26] Guinée P. A. Jansen W. H. Agterberg C. M. (1976). Detection of the K99 antigen by means of agglutination and immunoelectrophoresis in *Escherichia coli* isolates from calves and its correlation with entertoxigenicity. Infect. Immun. 13, 1369–1377. 773832 10.1128/iai.13.5.1369-1377.1976 PMC420767

[B27] Han M.-J. Lee S. Y. (2006). The *Escherichia coli* proteome: past, present, and future prospects. Microbiol. Mol. Biol. Rev. 70, 362–439. 10.1128/MMBR.00036-05 16760308 PMC1489533

[B28] Hoey D. E. E. Currie C. Else R. W. Nutikka A. Lingwood C. A. Gally D. L.. (2002). Expression of receptors for verotoxin 1 from *Escherichia coli* O157 on bovine intestinal epithelium. J. Med. Microbiol. 51, 143–149. 11865842 10.1099/0022-1317-51-2-143

[B29] Jensen C. O. (1903). Om den infektiese Kalverdiarrhoe og dens Aarsag. Maanedsskrift Dyrlaeger 4, 140–145.

[B30] Kaper J. B. Nataro J. P. Mobley H. L. (2004). Pathogenic *Escherichia coli*. Nat. Rev. Microbiol. 2, 123–140. 10.1038/nrmicro818 15040260

[B31] Konowalchuk J. Speirs J. I. Stavric S. (1977). Vero response to a cytotoxin of *Escherichia coli*. Infect. Immun. 18, 775–779. 338490 10.1128/iai.18.3.775-779.1977 PMC421302

[B32] Lacher D. W. Gangiredla J. Jackson S. A. Elkins C. A. Feng P. C. H. (2014). A novel microarray design for molecular serotyping of Shiga toxin-producing *Escherichia coli* isolated from fresh produce. Appl. Environ. Microbiol. 80, 4677–4682. 10.1128/AEM.01049-14 24837388 PMC4148803

[B33] Lai Y. Rosenshine I. Leong J. M. Frankel G. (2013). Intimate host attachment: enteropathogenic and enterohaemorrhagic *Escherichia coli*. Cell. Microbiol. 15, 1796–1808. 10.1111/cmi.12179 23927593 PMC4036124

[B34] Lorenz S. C. Son I. Maounounen-Laasri A. Lin A. Fischer M. Kase J. A. (2013). Prevalence of hemolysin genes and comparison of ehxA subtype patterns in Shiga toxin-producing *Escherichia coli* (STEC) and non-STEC strains from clinical, food, and animal sources. Appl. Environ. Microbiol. 79, 6301–6311. 10.1128/AEM.02200-13 23934487 PMC3811216

[B35] Menge C. Wieler L. H. Schlapp T. Baljer G. (1999). Shiga toxin 1 from *Escherichia coli* blocks activation and proliferation of bovine lymphocyte subpopulations *in vitro*. Infect. Immun. 67, 2209–2217. 10225876 10.1128/iai.67.5.2209-2217.1999 PMC115959

[B36] Moher D. Liberati A. Tetzlaff J. Altman D. G. The PRISMA Group. (2009). Preferred reporting items for systematic reviews and meta-analyses: the PRISMA statement. PLoS Med. 6:e1000097. 10.1371/journal.pmed.1000097 19621072 PMC2707599

[B37] Moon H. W. Bunn T. O. (1993). Vaccines for preventing enterotoxigenic *Escherichia coli* infections in farm animals. Vaccine 11, 213–200. 10.1016/0264-410X(93)90020-X 8094931 PMC7130883

[B38] Moseley S. L. Huq I. Alim A. R. So M. Samadpour-Motalebi M. Falkow S. (1980). Detection of enterotoxigenic *Escherichia coli* by DNA colony hybridization. J. Infect. Dis. 142, 892–898. 10.1093/infdis/142.6.892 7007526

[B39] Nagy B. Fekete P. Z. (1999). Enterotoxigenic *Escherichia coli* (ETEC) in farm animals. Vet. Res. 30, 259–284. 10367358

[B40] Nagy B. Fekete P. Z. (2005). Enterotoxigenic *Escherichia coli* in veterinary medicine. Int. J. Med. Microbiol. 295, 443–454. 10.1016/j.ijmm.2005.07.003 16238018

[B41] Nguyen Y. Sperandio V. (2012). Enterohemorrhagic *E. coli (EHEC)* pathogenesis. Front. Cell. Infect. Microbiol. 2:90. 10.3389/fcimb.2012.00090 22919681 PMC3417627

[B42] Orden J. A. Domínguez-Bernal G. Martínez-Pulgarín S. Blanco M. Blanco J. E. Mora A.. (2007). Necrotoxigenic *Escherichia coli* from sheep and goats produce a new type of cytotoxic necrotizing factor (CNF3) associated with the eae and ehxA genes. Int. Microbiol. 10, 47–55. 10.2436/20.1501.01.7 17407060

[B43] Orskov I. Orskov F. Jann B. Jann K. (1977). Serology, chemistry, and genetics of O and K antigens of *Escherichia coli*. Bacteriol. Rev. 41, 667–710. 334154 10.1128/br.41.3.667-710.1977 PMC414020

[B44] Orskov I. Orskov F. Smith H. W. Sojka W. J. (1975). The establishment of K99, a thermolabile, transmissible *Escherichia coli* K antigen, previously called “Kco,” possessed by calf and lamb enteropathogenic strains. Acta Pathol. Microbiol. Scand. B. 83, 31–36. 1093354 10.1111/j.1699-0463.1975.tb00066.x

[B45] Ortiz-Pelaez A. Pritchard D. G. Pfeiffer D. U. Jones E. Honeyman P. Mawdsley J. J. (2008). Calf mortality as a welfare indicator on British cattle farms. Vet. J. 176, 177–181. 10.1016/j.tvjl.2007.02.006 17408994

[B46] Pruimboom-Brees I. M. Morgan T. W. Ackermann M. R. Nystrom E. D. Samuel J. E. Cornick N. A.. (2000). Cattle lack vascular receptors for *Escherichia coli* O157:H7 Shiga toxins. Proc. Natl. Acad. Sci. U.S.A. 97, 10325–10329. 10.1073/pnas.190329997 10973498 PMC27023

[B47] R Development Core Team (2013). R: A Language and Environment for Statistical Computing. Vienna: R Foundation for Statistical Computing Available online at: http://www.r-project.org

[B48] Rödiger S. Schierack P. Böhm A. Nitschke J. Berger I. Frömmel U.. (2013). A highly versatile microscope imaging technology platform for the multiplex real-time detection of biomolecules and autoimmune antibodies. Adv. Biochem. Eng. Biotechnol. 133, 35–74. 10.1007/10_2011_132 22437246

[B49] Römer A. Wieler L. H. Schierack P. (2012). Analyses of intestinal commensal *Escherichia coli* strains from wild boars suggest adaptation to conventional pig production conditions. Vet. Microbiol. 161, 122–129. 10.1016/j.vetmic.2012.07.009 22857976

[B50] Ruan X. Crupper S. S. Schultz B. D. Robertson D. C. Zhang W. (2012). *Escherichia coli* expressing EAST1 toxin did not cause an increase of cAMP or cGMP levels in cells, and no diarrhea in 5-day old Gnotobiotic Pigs. PLoS ONE 7:e43203. 10.1371/journal.pone.0043203 22905235 PMC3419656

[B51] Schierack P. Steinrück H. Kleta S. Vahjen W. (2006). Virulence factor gene profiles of *Escherichia coli* isolates from clinically healthy pigs. Appl. Environ. Microbiol. 72, 6680–6686. 10.1128/AEM.02952-05 17021219 PMC1610323

[B52] Schierack P. Weinreich J. Ewers C. Tachu B. Nicholson B. Barth S. (2011). Hemolytic porcine intestinal *Escherichia coli* without virulence-associated genes typical of intestinal pathogenic *E. coli*. Appl. Environ. Microbiol. 77, 8451–8455. 10.1128/AEM.05289-11 21965399 PMC3233061

[B53] Servin A. L. (2005). Pathogenesis of Afa/Dr diffusely adhering *Escherichia coli*. Clin. Microbiol. Rev. 18, 264–292. 10.1128/CMR.18.2.264-292.2005 15831825 PMC1082796

[B54] Smith H. W. Halls S. (1967). Observations by the ligated intestinal segment and oral inoculation methods on *Escherichia coli* infections in pigs, calves, lambs and rabbits. J. Pathol. 93, 499–529. 10.1002/path.1700930211 4861400

[B55] Smith T. Orcutt M. L. (1925). The becteriology of the intestinal tract of young calves with special referenceto the early diarrhea (“scours”). J. Exp. Med. 41, 89–106. 10.1084/jem.41.1.89 19868978 PMC2130927

[B56] Smith W. W. (1962). Observations on the ætiology of neonatal diarrhœa (scours) in calves. J. Pathol. 84, 147–168. 10.1002/path.1700840117 13914264

[B57] Sorek R. Cossart P. (2010). Prokaryotic transcriptomics: a new view on regulation, physiology and pathogenicity. Nat. Rev. Genet. 11, 9–16. 10.1038/nrg2695 19935729

[B58] Stroup D. F. Berlin J. A. Morton S. C. Olkin I. Williamson G. D. Rennie D.. (2000). Meta-analysis of observational studies in epidemiology: a proposal for reporting. Meta-analysis of observational studies in Epidemiology (MOOSE) group. JAMA 283, 2008–2012. 10.1001/jama.283.15.2008 10789670

[B59] Titze C. Weichel A. (1908). Die ätiologie der kälberruhr. Berlin. Tierärtzl. Wochschr. 26, 181–192.

[B60] Weintraub A. (2007). Enteroaggregative *Escherichia coli*: epidemiology, virulence and detection. J. Med. Microbiol. 56, 4–8. 10.1099/jmm.0.46930-0 17172509

[B61] Wickham H. (2010). A layered grammar of graphics. J. Comput. Graph. Stat. 19, 3–28 10.1198/jcgs.2009.07098

