# Supplementary material for: A systematic review and meta-analysis of the epidemiology of pathogenic Escherichia coli of calves and the role of calves as reservoirs for human pathogenic E. coli
Source: Front Cell Infect Microbiol. 2015 Mar 12;5:23. doi: 10.3389/fcimb.2015.00023 (PMC4357325; doi:10.3389/fcimb.2015.00023)
Supplement: Supplementary file 1 [file DataSheet1.DOCX]

***Supplementary Material***

**A systematic review and meta-analysis of the epidemiology of pathogenic *Escherichia coli* of calves and the role of calves as reservoirs for human pathogenic *E. coli*.**

Rafał Kolenda^1^, Michał Burdukiewicz^2^, Peter Schierack^1,^*

Brandenburg University of Technology Cottbus– Senftenberg, Faculty of Natural Sciences, Großenhainer Str. 57, D-01968, Senftenberg, Germany^1^University of Wrocław, Faculty of Biotechnology, Department of Genomics, ul. Fryderyka Joliot-Curie 14a, 50-383 Wrocław, Poland^2^

* Correspondence:

Peter Schierack, Faculty of Natural Sciences, Brandenburg University of Technology Cottbus– Senftenberg, Großenhainer Str. 57, D-01968, Senftenberg, Germany

peter.schierack@hs-lausitz.de

**Keywords: calves, diarrhea, ETEC, EPEC, STEC, EHEC, systematic review, meta-analysis**

1. **Supplementary Data**

## Suplementary File 1. PRISMA 2009 Checklist

| **Section/topic** | **#** | **Checklist item** | **Reported on page #** |
| --- | --- | --- | --- |
| **TITLE** | | |  |
| Title | 1 | Identify the report as a systematic review, meta-analysis, or both. | 1 |
| **ABSTRACT** | | |  |
| Structured summary | 2 | Provide a structured summary including, as applicable: background; objectives; data sources; study eligibility criteria, participants, and interventions; study appraisal and synthesis methods; results; limitations; conclusions and implications of key findings; systematic review registration number. | 2 |
| **INTRODUCTION** | | |  |
| Rationale | 3 | Describe the rationale for the review in the context of what is already known. | 3 |
| Objectives | 4 | Provide an explicit statement of questions being addressed with reference to participants, interventions, comparisons, outcomes, and study design (PICOS). | 6 |
| **METHODS** | | |  |
| Protocol and registration | 5 | Indicate if a review protocol exists, if and where it can be accessed (e.g., Web address), and, if available, provide registration information including registration number. | 6 |
| Eligibility criteria | 6 | Specify study characteristics (e.g., PICOS, length of follow-up) and report characteristics (e.g., years considered, language, publication status) used as criteria for eligibility, giving rationale. | 6 |
| Information sources | 7 | Describe all information sources (e.g., databases with dates of coverage, contact with study authors to identify additional studies) in the search and date last searched. | 6 |
| Search | 8 | Present full electronic search strategy for at least one database, including any limits used, such that it could be repeated. | 6 |
| Study selection | 9 | State the process for selecting studies (i.e., screening, eligibility, included in systematic review, and, if applicable, included in the meta-analysis). | 7 |
| Data collection process | 10 | Describe method of data extraction from reports (e.g., piloted forms, independently, in duplicate) and any processes for obtaining and confirming data from investigators. | 7 |
| Data items | 11 | List and define all variables for which data were sought (e.g., PICOS, funding sources) and any assumptions and simplifications made. | 8 |
| Risk of bias in individual studies | 12 | Describe methods used for assessing risk of bias of individual studies (including specification of whether this was done at the study or outcome level), and how this information is to be used in any data synthesis. |  |
| Summary measures | 13 | State the principal summary measures (e.g., risk ratio, difference in means). | 8 |
| Synthesis of results | 14 | Describe the methods of handling data and combining results of studies, if done, including measures of consistency (e.g., I^2^) for each meta-analysis. | 8 |

| **Section/topic** | **#** | **Checklist item** | **Reported on page #** |
| --- | --- | --- | --- |
| Risk of bias across studies | 15 | Specify any assessment of risk of bias that may affect the cumulative evidence (e.g., publication bias, selective reporting within studies). |  |
| Additional analyses | 16 | Describe methods of additional analyses (e.g., sensitivity or subgroup analyses, meta-regression), if done, indicating which were pre-specified. | 8 |
| **RESULTS** | | |  |
| Study selection | 17 | Give numbers of studies screened, assessed for eligibility, and included in the review, with reasons for exclusions at each stage, ideally with a flow diagram. | 8 |
| Study characteristics | 18 | For each study, present characteristics for which data were extracted (e.g., study size, PICOS, follow-up period) and provide the citations. | 9 |
| Risk of bias within studies | 19 | Present data on risk of bias of each study and, if available, any outcome level assessment (see item 12). |  |
| Results of individual studies | 20 | For all outcomes considered (benefits or harms), present, for each study: (a) simple summary data for each intervention group (b) effect estimates and confidence intervals, ideally with a forest plot. | 9-11 |
| Synthesis of results | 21 | Present results of each meta-analysis done, including confidence intervals and measures of consistency. | 9-11 |
| Risk of bias across studies | 22 | Present results of any assessment of risk of bias across studies (see Item 15). |  |
| Additional analysis | 23 | Give results of additional analyses, if done (e.g., sensitivity or subgroup analyses, meta-regression [see Item 16]). | 9-11 |
| **DISCUSSION** | | |  |
| Summary of evidence | 24 | Summarize the main findings including the strength of evidence for each main outcome; consider their relevance to key groups (e.g., healthcare providers, users, and policy makers). | 12-14 |
| Limitations | 25 | Discuss limitations at study and outcome level (e.g., risk of bias), and at review-level (e.g., incomplete retrieval of identified research, reporting bias). | 14 |
| Conclusions | 26 | Provide a general interpretation of the results in the context of other evidence, and implications for future research. | 14 |
| **FUNDING** | | |  |
| Funding | 27 | Describe sources of funding for the systematic review and other support (e.g., supply of data); role of funders for the systematic review. | 15 |

*From:*  Moher D, Liberati A, Tetzlaff J, Altman DG, The PRISMA Group (2009). Preferred Reporting Items for Systematic Reviews and Meta-Analyses: The PRISMA Statement. PLoS Med 6(6): e1000097. doi:10.1371/journal.pmed1000097

## File S2 List of publications included in systematic review and meta-analysis

1. Achá, S. J., Kühn, I., Jonsson, P., Mbazima, G., Katouli, M., and Möllby, R. (2004). Studies on calf diarrhoea in Mozambique: prevalence of bacterial pathogens. *Acta Vet. Scand.* 45, 27–36.
2. Aidar, L., Penteado, A. S., Trabulsi, L. R., Blanco, J. E., Blanco, M., Blanco, J., and Pestana de Castro, A. F. (2000). Subtypes of intimin among non-toxigenic *Escherichia coli* from diarrheic calves in Brazil. *Can. J. Vet. Res.* 64, 15–20.
3. Aidar-Ugrinovich, L., Blanco, J., Blanco, M., Blanco, J. E., Leomil, L., Dahbi, G., Mora, A., Onuma, D. L., Silveira, W. D., and Pestana de Castro, A. F. (2007). Serotypes, virulence genes, and intimin types of Shiga toxin-producing *Escherichia coli* (STEC) and enteropathogenic *E. coli* (EPEC) isolated from calves in São Paulo, Brazil. *Int. J. Food Microbiol.* 115, 297–306. doi:10.1016/j.ijfoodmicro.2006.10.046.
4. Andrade, G. I., Coura, F. M., Santos, E. L. S., Ferreira, M. G., Galinari, G. C. F., Facury Filho, E. J., de Carvalho, A. U., Lage, A. P., and Heinemann, M. B. (2012). Identification of virulence factors by multiplex PCR in *Escherichia coli* isolated from calves in Minas Gerais, Brazil. *Trop Anim Health Prod* 44, 1783–1790. doi:10.1007/s11250-012-0139-8.
5. Arya, G., Roy, A., Choudhary, V., Yadav, M. M., and Joshi, C. G. (2008). Serogroups, atypical biochemical characters, colicinogeny and antibiotic resistance pattern of Shiga toxin-producing *Escherichia coli* isolated from diarrhoeic calves in Gujarat, India. *Zoonoses Public Health* 55, 89–98. doi:10.1111/j.1863-2378.2007.01093.x.
6. Bardiau, M., Labrozzo, S., and Mainil, J. G. (2009). Putative Adhesins of Enteropathogenic and Enterohemorrhagic *Escherichia coli* of Serogroup O26 Isolated from Humans and Cattle. *J Clin Microbiol* 47, 2090–2096. doi:10.1128/JCM.02048-08.
7. Barigye, R., Gautam, A., Piche, L. M., Schaan, L. P., Krogh, D. F., and Olet, S. (2012). Prevalence and antimicrobial susceptibility of virulent and avirulent multidrug-resistant *Escherichia coli* isolated from diarrheic neonatal calves. *Am. J. Vet. Res.* 73, 1944–1950. doi:10.2460/ajvr.73.12.1944.
8. Bartels, C. J. M., Holzhauer, M., Jorritsma, R., Swart, W. A. J. M., and Lam, T. J. G. M. (2010). Prevalence, prediction and risk factors of enteropathogens in normal and non-normal faeces of young Dutch dairy calves. *Prev. Vet. Med.* 93, 162–169. doi:10.1016/j.prevetmed.2009.09.020.
9. Bertin, Y., Girardeau, J.-P., Darfeuille-Michaud, A., and Martin, C. (2000). Epidemiological Study of pap Genes among Diarrheagenic or Septicemic *Escherichia coli* Strains Producing CS31A and F17  Adhesins and Characterization of Pap31A Fimbriae. *J Clin Microbiol* 38, 1502–1509.
10. Björkman, C., Svensson, C., Christensson, B., and de Verdier, K. (2003). *Cryptosporidium parvum* and *Giardia intestinalis* in calf diarrhoea in Sweden. *Acta Vet. Scand.* 44, 145–152.
11. Blanco, M., Blanco, J., Blanco, J. E., González, E. A., Gomes, T. A. T., Zerbini, L. F., Yano, T., and de Castro, A. F. P. (1994). Genes coding for Shiga-like toxins in bovine verotoxin-producing *Escherichia coli* (VTEC) strains belonging to different O:K:H serotypes. *Veterinary Microbiology* 42, 105–110. doi:10.1016/0378-1135(94)90010-8.
12. Blanco, M., Blanco, J. E., Blanco, J., Gonzalez, E. A., Mora, A., Prado, C., Fernández, L., Rio, M., Ramos, J., and Alonso, M. P. (1996). Prevalence and characteristics of *Escherichia coli* serotype O157:H7 and other verotoxin-producing *E. coli* in healthy cattle. *Epidemiol. Infect.* 117, 251–257.
13. Blanco, M., Blanco, J. E., Blanco, J., Mora, A., Prado, C., Alonso, M. P., Mouriño, M., Madrid, C., Balsalobre, C., and Juárez, A. (1997). Distribution and characterization of faecal verotoxin-producing *Escherichia coli* (VTEC) isolated from healthy cattle. *Vet. Microbiol.* 54, 309–319.
14. Blanco, M., Blanco, J. E., Mora, A., and Blanco, J. (1998). Prevalence and characteristics of necrotoxigenic *Escherichia coli* CNF1+ and CNF2+ in healthy cattle. *Research in Microbiology* 149, 47–53. doi:10.1016/S0923-2508(97)83623-6.
15. Bonardi, S., Maggi, E., Bottarelli, A., Pacciarini, M. L., Ansuini, A., Vellini, G., Morabito, S., and Caprioli, A. (1999). Isolation of Verocytotoxin-producing *Escherichia coli* O157:H7 from cattle at slaughter in Italy. *Vet. Microbiol.* 67, 203–211.
16. Van Bost, S., Bâbe, M. H., Jacquemin, E., and Mainil, J. (2001). Characteristics of necrotoxigenic *Escherichia coli* isolated from septicemic and diarrheic calves between 1958 and 1970. *Vet. Microbiol.* 82, 311–320.
17. Al-Charrakh, A., and Al-Muhana, A. (2010). Prevalence of Verotoxin-Producing *Escherichia coli* (VTEC) in a survey of dairy cattle in Najaf, Iraq. *Iran J Microbiol* 2, 130–136.
18. China, B., Jacquemin, E., Devrin, A. C., Pirson, V., and Mainil, J. (1999). Heterogeneity of the eae genes in attaching/effacing *Escherichia coli* from cattle: comparison with human strains. *Res. Microbiol.* 150, 323–332.
19. China, B., Pirson, V., and Mainil, J. (1998). Prevalence and molecular typing of attaching and effacing *Escherichia coli* among calf populations in Belgium. *Vet. Microbiol.* 63, 249–259.
20. China, B., Pirson, V., and Mainil, J. (1996). Typing of bovine attaching and effacing *Escherichia coli* by multiplex in vitro amplification of virulence-associated genes. *Appl Environ Microbiol* 62, 3462–3465.
21. Chinen, I., Otero, J. L., Miliwebsky, E. S., Roldán, M. L., Baschkier, A., Chillemi, G. M., Nóboli, C., Frizzo, L., and Rivas, M. (2003). Isolation and characterisation of Shiga toxin-producing *Escherichia coli* O157:H7 from calves in Argentina. *Research in Veterinary Science* 74, 283–286. doi:10.1016/S0034-5288(02)00192-3.
22. Cho, S., Diez-Gonzalez, F., Fossler, C. P., Wells, S. J., Hedberg, C. W., Kaneene, J. B., Ruegg, P. L., Warnick, L. D., and Bender, J. B. (2006). Prevalence of shiga toxin-encoding bacteria and shiga toxin-producing *Escherichia coli* isolates from dairy farms and county fairs. *Vet. Microbiol.* 118, 289–298. doi:10.1016/j.vetmic.2006.07.021.
23. Cho, Y.-I., Han, J.-I., Wang, C., Cooper, V., Schwartz, K., Engelken, T., and Yoon, K.-J. (2013). Case-control study of microbiological etiology associated with calf diarrhea. *Vet. Microbiol.* 166, 375–385. doi:10.1016/j.vetmic.2013.07.001.
24. Cho, Y.-I., Kim, W.-I., Liu, S., Kinyon, J. M., and Yoon, K. J. (2010). Development of a panel of multiplex real-time polymerase chain reaction assays for simultaneous detection of major agents causing calf diarrhea in feces. *J. Vet. Diagn. Invest.* 22, 509–517.
25. Cho, Y.-I., Sun, D., Cooper, V., Dewell, G., Schwartz, K., and Yoon, K.-J. (2012). Evaluation of a commercial rapid test kit for detecting bovine enteric pathogens in feces. *J. Vet. Diagn. Invest.* 24, 559–562. doi:10.1177/1040638712440997.
26. Cobbold, R., and Desmarchelier, P. (2001). Characterisation and clonal relationships of Shiga-toxigenic *Escherichia coli* (STEC) isolated from Australian dairy cattle. *Vet. Microbiol.* 79, 323–335.
27. Contrepois, M., Bertin, Y., Pohl, P., Picard, B., and Girardeau, J.-P. (1998). A study of relationships among F17 a producing enterotoxigenic and non-enterotoxigenic *Escherichia coli* strains isolated from diarrheic calves. *Veterinary Microbiology* 64, 75–81. doi:10.1016/S0378-1135(98)00253-3.
28. Cookson, A. L., Taylor, S. C. S., Bennett, J., Thomson-Carter, F., and Attwood, G. T. (2006). Serotypes and analysis of distribution of Shiga toxin producing *Escherichia coli* from cattle and sheep in the lower North Island, New Zealand. *N Z Vet J* 54, 78–84. doi:10.1080/00480169.2006.36616.
29. Cristancho, L., Johnson, R. P., McEwen, S. A., and Gyles, C. L. (2008). *Escherichia coli* O157:H7 and other Shiga toxin-producing *E. coli* in white veal calves. *Vet. Microbiol.* 126, 200–209. doi:10.1016/j.vetmic.2007.06.012.
30. Das, S. C., Khan, A., Panja, P., Datta, S., Sikdar, A., Yamasaki, S., Takeda, Y., Bhattacharya, S. K., Ramamurthy, T., and Nair, G. B. (2005). Dairy farm investigation on Shiga toxin-producing *Escherichia coli* (STEC) in Kolkata, India with emphasis on molecular characterization. *Epidemiol. Infect.* 133, 617–626.
31. Dastmalchi, S. H., and Ayremlou, N. (2012). Characterization of Shiga toxin-producing *Escherichia coli* (STEC) in feces of healthy and diarrheic calves in Urmia region, Iran. *Iran J Microbiol* 4, 63–69.
32. Donaldson, S. C., Straley, B. A., Hegde, N. V., Sawant, A. A., DebRoy, C., and Jayarao, B. M. (2006). Molecular Epidemiology of Ceftiofur-Resistant *Escherichia coli* Isolates from Dairy Calves. *Appl Environ Microbiol* 72, 3940–3948. doi:10.1128/AEM.02770-05.
33. Ellens, D. J., de Leeuw, P. W., and Rozemond, H. (1979). Detection of the K99 antigen of *Escherichia coli* in calf faeces by enzyme-linked immunosorbent assay (ELISA). *Vet Q* 1, 169–175. doi:10.1080/01652176.1979.9693743.
34. Evans, J., Knight, H. I., Smith, A. W., Pearce, M. C., Hall, M., Foster, G., Low, J. C., and Gunn, G. J. (2008). Cefixime-tellurite rhamnose MacConkey agar for isolation of Vero cytotoxin-producing *Escherichia coli* serogroup O26 from Scottish cattle and sheep faeces. *Lett. Appl. Microbiol.* 47, 148–152. doi:10.1111/j.1472-765X.2008.02402.x.
35. Ewers, C., Schüffner, C., Weiss, R., Baljer, G., and Wieler, L. H. (2004). Molecular characteristics of *Escherichia coli* serogroup O78 strains isolated from diarrheal cases in bovines urge further investigations on their zoonotic potential. *Mol Nutr Food Res* 48, 504–514. doi:10.1002/mnfr.200400063.
36. Fecteau, G., Fairbrother, J. M., Higgins, R., Van Metre, D. C., Paré, J., Smith, B. P., Holmberg, C. A., and Jang, S. (2001). Virulence factors in *Escherichia coli* isolated from the blood of bacteremic neonatal calves. *Vet. Microbiol.* 78, 241–249.
37. Fernández, D., Rodríguez, E. M., Arroyo, G. H., Padola, N. L., and Parma, A. E. (2009). Seasonal variation of Shiga toxin-encoding genes (stx) and detection of *E. coli* O157 in dairy cattle from Argentina. *J. Appl. Microbiol.* 106, 1260–1267. doi:10.1111/j.1365-2672.2008.04088.x.
38. Fernández, D., Sanz, M. E., Parma, A. E., and Padola, N. L. (2012). Short communication: characterization of Shiga toxin-producing *Escherichia coli* isolated from newborn, milk-fed, and growing calves in Argentina. *J. Dairy Sci.* 95, 5340–5343. doi:10.3168/jds.2011-5140.
39. De la Fuente, R., García, A., Ruiz-Santa-Quiteria, J. A., Luzón, M., Cid, D., García, S., Orden, J. A., and Gómez-Bautista, M. (1998). Proportional morbidity rates of enteropathogens among diarrheic dairy calves in central Spain. *Prev. Vet. Med.* 36, 145–152.
40. De la Fuente, R., Luzón, M., Ruiz-Santa-Quiteria, J. A., García, A., Cid, D., Orden, J. A., García, S., Sanz, R., and Gómez-Bautista, M. (1999). *Cryptosporidium* and concurrent infections with other major enterophatogens in 1 to 30-day-old diarrheic dairy calves in central Spain. *Vet. Parasitol.* 80, 179–185.
41. Ganaba, R., Bigras-Poulin, M., Fairbrother, J. M., and Belanger, D. (1995). Importance of *Escherichia coli* in young beef calves from northwestern Quebec. *Can J Vet Res* 59, 20–25.
42. García, A., Ruiz-Santa-Quiteria, J. A., Orden, J. A., Cid, D., Sanz, R., Gómez-Bautista, M., and de la Fuente, R. (2000). Rotavirus and concurrent infections with other enteropathogens in neonatal diarrheic dairy calves in Spain. *Comp. Immunol. Microbiol. Infect. Dis.* 23, 175–183.
43. Ghanbarpour, R., and Oswald, E. (2009). Characteristics and virulence genes of *Escherichia coli* isolated from septicemic calves in southeast of Iran. *Trop Anim Health Prod* 41, 1091–1099. doi:10.1007/s11250-008-9289-0.
44. Giammanco, G. M., Pignato, S., Grimont, F., Grimont, P. A. D., Caprioli, A., Morabito, S., and Giammanco, G. (2002). Characterization of Shiga Toxin-Producing *Escherichia coli* O157:H7 Isolated in Italy and in France. *J. Clin. Microbiol.* 40, 4619–4624. doi:10.1128/JCM.40.12.4619-4624.2002.
45. Girardeau, J. P., Lalioui, L., Said, A. M. O., De Champs, C., and Le Bouguénec, C. (2003). Extended virulence genotype of pathogenic *Escherichia coli* isolates carrying the afa-8 operon: evidence of similarities between isolates from humans and animals with extraintestinal infections. *J. Clin. Microbiol.* 41, 218–226.
46. Guinée, P. A., Jansen, W. H., and Agterberg, C. M. (1976). Detection of the K99 antigen by means of agglutination and immunoelectrophoresis in *Escherichia coli* isolates from calves and its correlation with entertoxigenicity. *Infect. Immun.* 13, 1369–1377.
47. Güler, L., Gündüz, K., and Ok, U. (2008). Virulence factors and antimicrobial susceptibility of *Escherichia coli* isolated from calves in Turkey. *Zoonoses Public Health* 55, 249–257. doi:10.1111/j.1863-2378.2008.01121.x.
48. Gulliksen, S. M., Jor, E., Lie, K. I., Hamnes, I. S., Løken, T., Akerstedt, J., and Osterås, O. (2009). Enteropathogens and risk factors for diarrhea in Norwegian dairy calves. *J. Dairy Sci.* 92, 5057–5066. doi:10.3168/jds.2009-2080.
49. Harnett, N. M., and Gyles, C. L. (1985). Enterotoxin plasmids in bovine and porcine enterotoxigenic *Escherichia coli* of O groups 9, 20, 64 and 101. *Can J Comp Med* 49, 79–87.
50. Holland, R. E., Wilson, R. A., Holland, M. S., Yuzbasiyan-Gurkan, V., Mullaney, T. P., and White, D. G. (1999). Characterization of eae+ *Escherichia coli* isolated from healthy and diarrheic calves. *Vet. Microbiol.* 66, 251–263.
51. Hur, J., Jeon, B. W., Kim, Y. J., Oh, I. G., and Lee, J. H. (2013). *Escherichia coli* isolates from calf diarrhea in Korea and their virulent genetic characteristics. *J. Vet. Med. Sci.* 75, 519–522.
52. Irshad, H., Cookson, A. L., Hotter, G., Besser, T. E., On, S. L. W., and French, N. P. (2012). Epidemiology of Shiga toxin-producing *Escherichia coli* O157 in very young calves in the North Island of New Zealand. *N Z Vet J* 60, 21–26. doi:10.1080/00480169.2011.627063.
53. Izzo, M. M., Kirkland, P. D., Mohler, V. L., Perkins, N. R., Gunn, A. A., and House, J. K. (2011). Prevalence of major enteric pathogens in Australian dairy calves with diarrhoea. *Aust. Vet. J.* 89, 167–173. doi:10.1111/j.1751-0813.2011.00692.x.
54. Janke, B. H., Francis, D. H., Collins, J. E., Libal, M. C., Zeman, D. H., and Johnson, D. D. (1989). Attaching and effacing *Escherichia coli* infections in calves, pigs, lambs, and dogs. *J. Vet. Diagn. Invest.* 1, 6–11.
55. Kang, S. J., Ryu, S. J., Chae, J. S., Eo, S. K., Woo, G. J., and Lee, J. H. (2004). Occurrence and characteristics of enterohemorrhagic *Escherichia coli* O157 in calves associated with diarrhoea. *Vet. Microbiol.* 98, 323–328. doi:10.1016/j.vetmic.2003.11.004.
56. Kobayashi, H., Miura, A., Hayashi, H., Ogawa, T., Endo, T., Hata, E., Eguchi, M., and Yamamoto, K. (2003). Prevalence and Characteristics of eae-Positive *Escherichia coli* from Healthy Cattle in Japan. *Appl Environ Microbiol* 69, 5690–5692. doi:10.1128/AEM.69.9.5690-5692.2003.
57. Lee, J. H., Hur, J., and Stein, B. D. (2008). Occurrence and characteristics of enterohemorrhagic *Escherichia coli* O26 and O111 in calves associated with diarrhea. *The Veterinary Journal* 176, 205–209. doi:10.1016/j.tvjl.2007.02.007.
58. Leomil, L., Aidar-Ugrinovich, L., Guth, B. E. C., Irino, K., Vettorato, M. P., Onuma, D. L., and de Castro, A. F. P. (2003). Frequency of Shiga toxin-producing *Escherichia coli* (STEC) isolates among diarrheic and non-diarrheic calves in Brazil. *Veterinary Microbiology* 97, 103–109. doi:10.1016/j.vetmic.2003.08.002.
59. Manna, S. K., Brahmane, M. P., Manna, C., Batabyal, K., and Das, R. (2006). Occurrence, virulence characteristics and antimicrobial resistance of *Escherichia coli* O157 in slaughtered cattle and diarrhoeic calves in West Bengal, India. *Lett. Appl. Microbiol.* 43, 405–409. doi:10.1111/j.1472-765X.2006.01975.x.
60. Masana, M. O., Leotta, G. A., Del Castillo, L. L., D’Astek, B. A., Palladino, P. M., Galli, L., Vilacoba, E., Carbonari, C., Rodríguez, H. R., and Rivas, M. (2010). Prevalence, characterization, and genotypic analysis of *Escherichia coli* O157:H7/NM from selected beef exporting abattoirs of Argentina. *J. Food Prot.* 73, 649–656.
61. M Askari Badouei, T. Z. S. (2010). Virulence gene profiles and intimin subtypes of Shiga toxin-producing *Escherichia coli* isolated from healthy and diarrhoeic calves. *The Veterinary record* 167, 858–61. doi:10.1136/vr.c4009.
62. Mechie, S. C., Chapman, P. A., and Siddons, C. A. (1997). A fifteen month study of *Escherichia coli* O157:H7 in a dairy herd. *Epidemiol. Infect.* 118, 17–25.
63. Mercado, E. C., Gioffré, A., Rodríguez, S. M., Cataldi, A., Irino, K., Elizondo, A. M., Cipolla, A. L., Romano, M. I., Malena, R., and Méndez, M. A. (2004). Non-O157 Shiga toxin-producing *Escherichia coli* isolated from diarrhoeic calves in Argentina. *J. Vet. Med. B Infect. Dis. Vet. Public Health* 51, 82–88. doi:10.1111/j.1439-0450.2004.00729.x.
64. Mercado, E. C., Rodríguez, S. M., D’Antuono, A. L., Cipolla, A. L., Elizondo, A. M., Rossetti, C. A., Malena, R., and Méndez, M. A. (2003). Occurrence and characteristics of CS31A antigen-producing *Escherichia* *coli* in calves with diarrhoea and septicaemia in Argentina. *J. Vet. Med. B Infect. Dis. Vet. Public Health* 50, 8–13.
65. Mills, K. W., and Tietze, K. L. (1984). Monoclonal antibody enzyme-linked immunosorbent assay for identification of K99-positive *Escherichia* *coli* isolates from calves. *J Clin Microbiol* 19, 498–501.
66. Myers, L. L., and Guinée, P. A. (1976). Occurrence and characteristics of enterotoxigenic *Escherichia* *coli* isolated from calves with diarrhea. *Infect. Immun.* 13, 1117–1119.
67. Nguyen, T. D., Vo, T. T., and Vu-Khac, H. (2011). Virulence factors in *Escherichia* *coli* isolated from calves with diarrhea in Vietnam. *J. Vet. Sci.* 12, 159–164.
68. Ok, M., Güler, L., Turgut, K., Ok, U., Sen, I., Gündüz, I. K., Birdane, M. F., and Güzelbekteş, H. (2009). The studies on the aetiology of diarrhoea in neonatal calves and determination of virulence gene markers of *Escherichia* *coli* strains by multiplex PCR. *Zoonoses Public Health* 56, 94–101. doi:10.1111/j.1863-2378.2008.01156.x.
69. Orden, J. A., Cid, D., Ruiz-Santa-Quiteria, J. A., García, S., Martínez, S., and de la Fuente, R. (2002). Verotoxin-producing *Escherichia* *coli* (VTEC), enteropathogenic *E. coli* (EPEC) and necrotoxigenic *E. coli* (NTEC) isolated from healthy cattle in Spain. *J. Appl. Microbiol.* 93, 29–35.
70. Orden, J. A., Cortés, C., Ruiz-Santa-quiteria, J. A., Martínez, S., and de la Fuente, R. (2005). Detection of the saa gene in verotoxin-producing *Escherichia* *coli* from ruminants. *J. Vet. Diagn. Invest.* 17, 65–67.
71. Orden, J. A., Ruiz-Santa-Quiteria, J. A., Cid, D., García, S., Sanz, R., and de la Fuente, R. (1998). Verotoxin-producing *Escherichia* *coli* (VTEC) and eae-positive non-VTEC in 1-30-days-old diarrhoeic dairy calves. *Vet. Microbiol.* 63, 239–248.
72. Osek, J. (2001). Characterization of necrotoxigenic *Escherichia* *coli* (NTEC) strains isolated from healthy calves in Poland. *J. Vet. Med. B Infect. Dis. Vet. Public Health* 48, 641–646.
73. Osek, J., Gallien, P., and Protz, D. (2000). Characterization of shiga toxin-producing *Escherichia* *coli* strains isolated from calves in Poland. *Comparative Immunology, Microbiology and Infectious Diseases* 23, 267–276. doi:10.1016/S0147-9571(00)00008-4.
74. Osek, J., and Winiarczyk, S. (2001). Prevalence of eae and shiga toxin genes among *Escherichia* *coli* strains isolated from healthy calves. *J. Vet. Med. B Infect. Dis. Vet. Public Health* 48, 67–72.
75. Osman, K. M., Mustafa, A. M., Elhariri, M., and Abdelhamed, G. S. (2013). The distribution of *Escherichia* *coli* serovars, virulence genes, gene association and combinations and virulence genes encoding serotypes in pathogenic *E. coli* recovered from diarrhoeic calves, sheep and goat. *Transbound Emerg Dis* 60, 69–78. doi:10.1111/j.1865-1682.2012.01319.x.
76. Oswald, E., de Rycke, J., Lintermans, P., van Muylem, K., Mainil, J., Daube, G., and Pohl, P. (1991). Virulence factors associated with cytotoxic necrotizing factor type two in bovine diarrheic and septicemic strains of *Escherichia* *coli*. *J. Clin. Microbiol.* 29, 2522–2527.
77. Pearce, M. C., Jenkins, C., Vali, L., Smith, A. W., Knight, H. I., Cheasty, T., Smith, H. R., Gunn, G. J., Woolhouse, M. E. J., Amyes, S. G. B., et al. (2004). Temporal shedding patterns and virulence factors of *Escherichia* *coli* serogroups O26, O103, O111, O145, and O157 in a cohort of beef calves and their dams. *Appl. Environ. Microbiol.* 70, 1708–1716.
78. Pereira, R. V. V., Santos, T. M. A., Bicalho, M. L., Caixeta, L. S., Machado, V. S., and Bicalho, R. C. (2011). Antimicrobial resistance and prevalence of virulence factor genes in fecal *Escherichia* *coli* of Holstein calves fed milk with and without antimicrobials. *J. Dairy Sci.* 94, 4556–4565. doi:10.3168/jds.2011-4337.
79. Rahn, K., Renwick, S. A., Johnson, R. P., Wilson, J. B., Clarke, R. C., Alves, D., McEwen, S., Lior, H., and Spika, J. (1997). Persistence of *Escherichia* *coli* O157:H7 in dairy cattle and the dairy farm environment. *Epidemiol. Infect.* 119, 251–259.
80. Raybould, T. J., Crouch, C. F., and Acres, S. D. (1987). Monoclonal antibody passive hemagglutination and capture enzyme-linked immunosorbent assays for direct detection and quantitation of F41 and K99 fimbrial antigens in enterotoxigenic *Escherichia* *coli*. *J. Clin. Microbiol.* 25, 278–284.
81. Salvarani, S., Tramuta, C., Nebbia, P., and Robino, P. (2012). Occurrence and functionality of cycle inhibiting factor, cytotoxic necrotising factors and cytolethal distending toxins in *Escherichia* *coli* isolated from calves and dogs in Italy. *Res. Vet. Sci.* 92, 372–377. doi:10.1016/j.rvsc.2011.04.019.
82. Samadpour, M., Liston, J., Ongerth, J. E., and Tarr, P. I. (1990). Evaluation of DNA probes for detection of Shiga-like-toxin-producing *Escherichia* *coli* in food and calf fecal samples. *Appl. Environ. Microbiol.* 56, 1212–1215.
83. Sandhu, K. S., Clarke, R. C., McFadden, K., Brouwer, A., Louie, M., Wilson, J., Lior, H., and Gyles, C. L. (1996). Prevalence of the eaeA gene in verotoxigenic *Escherichia* *coli* strains from dairy cattle in Southwest Ontario. *Epidemiol. Infect.* 116, 1–7.
84. Saridakis, H. O., El Gared, S. A., Vidotto, M. C., and Guth, B. E. C. (1997). Virulence properties of *Escherichia* *coli* strains belonging to enteropathogenic (EPEC) serogroups isolated from calves with diarrhea. *Veterinary Microbiology* 54, 145–153. doi:10.1016/S0378-1135(96)01278-3.
85. Scotland, S. M., Willshaw, G. A., Smith, H. R., and Rowe, B. (1990). Properties of strains of *Escherichia* *coli* O26:H11 in relation to their enteropathogenic or enterohemorrhagic classification. *J. Infect. Dis.* 162, 1069–1074.
86. Shabana, I. I., Zaraket, H., and Suzuki, H. (2013). Molecular studies on diarrhea-associated *Escherichia* *coli* isolated from humans and animals in Egypt. *Vet. Microbiol.* 167, 532–539. doi:10.1016/j.vetmic.2013.08.014.
87. Shaw, D. J., Jenkins, C., Pearce, M. C., Cheasty, T., Gunn, G. J., Dougan, G., Smith, H. R., Woolhouse, M. E. J., and Frankel, G. (2004). Shedding patterns of verocytotoxin-producing *Escherichia* *coli* strains in a cohort of calves and their dams on a Scottish beef farm. *Appl. Environ. Microbiol.* 70, 7456–7465. doi:10.1128/AEM.70.12.7456-7465.2004.
88. Shinagawa, K., Kanehira, M., Omoe, K., Matsuda, I., Hu, D., Widiasih, D. A., and Sugii, S. (2000). Frequency of Shiga toxin-producing *Escherichia* *coli* in cattle at a breeding farm and at a slaughterhouse in Japan. *Vet. Microbiol.* 76, 305–309.
89. Shin, S. J., Chang, Y.-F., Timour, M., Lauderdale, T.-L., and Lein, D. H. (1994). Hybridization of clinical *Escherichia* *coli* isolates from calves and piglets in New York State with gene probes for enterotoxins (STaP, STb, LT), Shiga-like toxins (SLT-1, SLT-II) and adhesion factors (K88, K99, F41, 987P). *Veterinary Microbiology* 38, 217–225. doi:10.1016/0378-1135(94)90003-5.
90. Silverlås, C., de Verdier, K., Emanuelson, U., Mattsson, J. G., and Björkman, C. (2010). *Cryptosporidium* infection in herds with and without calf diarrhoeal problems. *Parasitol. Res.* 107, 1435–1444. doi:10.1007/s00436-010-2020-x.
91. Sisti, M., Benedetti, C., Lonzi, A., Schiavano, G. F., Pianetti, A., Romanini, I., and Bruscolini, F. (2004). Isolation of *Escherichia* *coli* O157 from human and bovine faeces in the Urbino area, Italy. *Int J Hyg Environ Health* 207, 577–583. doi:10.1078/1438-4639-00330.
92. Thompson, H. P., Dooley, J. S. G., Kenny, J., McCoy, M., Lowery, C. J., Moore, J. E., and Xiao, L. (2007). Genotypes and subtypes of *Cryptosporidium* spp. in neonatal calves in Northern Ireland. *Parasitol. Res.* 100, 619–624. doi:10.1007/s00436-006-0305-x.
93. Torsein, M., Lindberg, A., Sandgren, C. H., Waller, K. P., Törnquist, M., and Svensson, C. (2011). Risk factors for calf mortality in large Swedish dairy herds. *Prev. Vet. Med.* 99, 136–147. doi:10.1016/j.prevetmed.2010.12.001.
94. Tutenel, A. V., Pierard, D., Van Hoof, J., Cornelis, M., and De Zutter, L. (2003). Isolation and molecular characterization of *Escherichia* *coli* O157 isolated from cattle, pigs and chickens at slaughter. *Int. J. Food Microbiol.* 84, 63–69.
95. Uber, A. P., Trabulsi, L. R., Irino, K., Beutin, L., Ghilardi, A. C. R., Gomes, T. A. T., Liberatore, A. M. A., de Castro, A. F. P., and Elias, W. P. (2006). Enteroaggregative *Escherichia* *coli* from humans and animals differ in major phenotypical traits and virulence genes. *FEMS Microbiol. Lett.* 256, 251–257. doi:10.1111/j.1574-6968.2006.00124.x.
96. Uhde, F. L., Kaufmann, T., Sager, H., Albini, S., Zanoni, R., Schelling, E., and Meylan, M. (2008). Prevalence of four enteropathogens in the faeces of young diarrhoeic dairy calves in Switzerland. *Vet. Rec.* 163, 362–366.
97. De Verdier, K., Nyman, A., Greko, C., and Bengtsson, B. (2012). Antimicrobial resistance and virulence factors in *Escherichia* *coli* from Swedish dairy calves. *Acta Vet. Scand.* 54, 2. doi:10.1186/1751-0147-54-2.
98. De Visser, N. A., Breukink, H. J., van Zijderveld, F. G., and de Leeuw, P. W. (1987). Enteric infections in veal calves: a longitudinal study on four veal calf units. *Vet Q* 9, 289–296. doi:10.1080/01652176.1987.9694116.
99. Waltner-Toews, D., Martin, S. W., and Meek, A. H. (1986). An epidemiological study of selected calf pathogens on Holstein dairy farms in southwestern Ontario. *Can J Vet Res* 50, 307–313.
100. Wani, S. A., Bhat, M. A., Samanta, I., Nishikawa, Y., and Buchh, A. S. (2003). Isolation and characterization of Shiga toxin-producing *Escherichia* *coli* (STEC) and enteropathogenic *Escherichia* *coli* (EPEC) from calves and lambs with diarrhoea in India. *Lett. Appl. Microbiol.* 37, 121–126.
101. Wani, S. A., Hussain, I., Nabi, A., Fayaz, I., and Nishikawa, Y. (2007). Variants of eae and stx genes of atypical enteropathogenic *Escherichia* *coli* and non-O157 Shiga toxin-producing *Escherichia* *coli* from calves. *Lett. Appl. Microbiol.* 45, 610–615. doi:10.1111/j.1472-765X.2007.02235.x.
102. Wieler, L. H., Schwanitz, A., Vieler, E., Busse, B., Steinrück, H., Kaper, J. B., and Baljer, G. (1998). Virulence properties of Shiga toxin-producing *Escherichia* *coli* (STEC) strains of serogroup O118, a major group of STEC pathogens in calves. *J. Clin. Microbiol.* 36, 1604–1607.
103. Wieler, L. H., Vieler, E., Erpenstein, C., Schlapp, T., Steinrück, H., Bauerfeind, R., Byomi, A., and Baljer, G. (1996). Shiga toxin-producing *Escherichia* *coli* strains from bovines: association of adhesion with carriage of eae and other genes. *J. Clin. Microbiol.* 34, 2980–2984.
104. Wilson, J. B., McEwen, S. A., Clarke, R. C., Leslie, K. E., Wilson, R. A., Waltner-Toews, D., and Gyles, C. L. (1992). Distribution and characteristics of verocytotoxigenic *Escherichia* *coli* isolated from Ontario dairy cattle. *Epidemiol Infect* 108, 423–439.
105. Younis, E. E., Ahmed, A. M., El-Khodery, S. A., Osman, S. A., and El-Naker, Y. F. I. (2009). Molecular screening and risk factors of enterotoxigenic *Escherichia* *coli* and *Salmonella* spp. in diarrheic neonatal calves in Egypt. *Res. Vet. Sci.* 87, 373–379. doi:10.1016/j.rvsc.2009.04.006.
106. Zhao, T., Doyle, M. P., Shere, J., and Garber, L. (1995). Prevalence of enterohemorrhagic *Escherichia* *coli* O157:H7 in a survey of dairy herds. *Appl. Environ. Microbiol.* 61, 1290–1293.
107. **Supplementary Figures and Tables**

## Suplementary Tables

**Supplementary Table 1. VAF names after unification naming convention**

| Name | Nomenclature from publications |
| --- | --- |
| F6 | 987P, F6 |
| aerobactin | aerobactin, iucD, iutA |
| afa | afa, afa E-VIII, afa/draBC, afa8e, afaI B-C |
| aaf | aggR, aggR, aggA, aggC, aafA, aafC |
| EAST | astA, EAST |
| bfp | bfpA, bfpB |
| cdt | cdt, cdtIII, cdtIV |
| cif | cif |
| CS31 | clpE/clpG, clpG, CS31, CS31a |
| CNF | cnf1, cnf2, cnf |
| F1845 | daaE, F1845 |
| eaeA | eae, intimin, eaeA |
| EAF | EAF |
| EFA1 | efa1 |
| hly | EHEC-hly, ehly, ehlyA, ehxA, hly |
| eibG | eibG |
| LTI | eltIa, LTI, LTp |
| ent | ent |
| escV | escV |
| esp | espB, espP, espA |
| STI | estIa, estIb, STAIP, STI, STA, STIh, STIp |
| etpD | etpD |
| F17 | F111, F17, F17a, F17b, F17c |
| F165 | F165 |
| F4 | F4, K88 |
| F41 | F41, F41-like |
| F5 | fanA, F5, K99 |
| fim | fim |
| fyuA | fyuA |
| H7 | H7 |
| hcp | hcpA |
| HPI | HPI |
| ibe | ibeA |
| iha | iha |
| inv | invE |
| ipa | ipaC, ipaH |
| iroN | iroN |
| katP | katP |
| kpsMII | kpsMII |
| ldaE | ldaE |
| LT | LT, lt |
| LTII | ltII, LTII |
| malX | malX(PAI) |
| modD | modD |
| pap | papAH, papC, papEF, papG |
| paa | paa |
| pic | pic |
| saa | saa |
| sfa | sfa, sfa/focDE |
| foc | sfa/focDE |
| shf | shf |
| STII | Stb, STII |
| Stx | stx |
| Stx1 | stx1, slt1, VT1, VTX1, VI |
| Stx2 | Stx2, slt2, VT2, VTX2, VII |
| tia | tia |
| toxB | toxB |
| traT | traT |
| uidA | uidA |
| F18 | F18 |
| aap | aap |

**Supplementary Table 2. Summary of all included publications.**

| ID Publication | Publication Year | First Author | Publication Name | Country Name | Isolation Year | Group | Isolates Tested | Number of VAF tested | VAF tested |
| --- | --- | --- | --- | --- | --- | --- | --- | --- | --- |
| 1 | 2013 | Osman | The Distribution of *Escherichia coli* Serovars, Virulence Genes, Gene Association and Combinations and Virulence Genes Encoding Serotypes in Pathogenic E. coli Recovered from Diarrhoeic Calves, Sheep and Goat | Egypt | 2009 | Diarrheic | 84 | 11 | eaeA, F17, F41, F5, hly, LTI, LTII, STI, STII, stx1, stx2 |
| 2 | 2013 | Shabana | Molecular studies on diarrhea-associated *Escherichia coli* isolated from humans and animals in Egypt | Egypt | 2008* | Diarrheic | 14 | 19 | aaf, bfp, cdt, CNF, eaeA, EAST, F17, F18, F1845, F4, F41, F5, hly, inv, LT, STI, STII, stx1, stx2 |
| 3 | 2013 | Cho | Case–control study of microbiological etiology associated with calf diarrhea | USA | 2010 | Diarrheic, Healthy | 444 | 1 | F5 |
| 4 | 2012 | Salvarani | Occurrence and functionality of cycle inhibiting factor, cytotoxic necrotising factors and cytolethal distending toxins in *Escherichia coli* isolated from calves and dogs in Italy | Italy | 2008 | Diarrheic, Healthy | 276 | 3 | cdt, cif, CNF |
| 5 | 2012 | Andrade | Identification of virulence factors by multiplex PCR in *Escherichia coli* isolated from calves in Minas Gerais, Brazil | Brazil | 2007* | Diarrheic, Healthy | 156 | 6 | eaeA, F41, F5, STI, stx1, stx2 |
| 6 | 2012 | Hur | *Escherichia coli* isolates from calf diarrhea in Korea and their virulent genetic characteristics | South Korea | 2007* | Diarrheic | 314 | 9 | Afa, CS31, eaeA, F17, F41, F5, STI, stx1, stx2 |
| 7 | 2012 | de Verdier | Antimicrobial resistance and virulence factors in *Escherichia coli* from Swedish dairy calves | Sweden | 2004 | Unknown | 94 | 26 | bfp, eaeA, EAST, esp, etpD, F18, F4, F41, F5, F6, fim, fliC, fyuA, hly, irp, katP, LT, STI, STII, stx1, stx2, terA, terC, terE, terW, terZ |
| 8 | 2012 | Fernández | Short communication: Characterization of Shiga toxin-producing *Escherichia coli* isolated from newborn, milk-fed, and growing calves in argentina | Argentina | 2006 | Healthy | 808 | 5 | eaeA, hly, saa, stx1, stx2 |
| 9 | 2012 | Cho | Evaluation of a commercial rapid test kit for detecting bovine enteric pathogens in feces | USA | 2010 | Diarrheic | 100 | 1 | F5 |
| 10 | 2012 | Irshad | Epidemiology of Shiga toxin-producing *Escherichia coli* O157 in very young calves in the North Island of New Zealand | New Zealand | 2008 | Mixed | 55 | 4 | eaeA, hly, stx1, stx2 |
| 11 | 2012 | Dastmalchi Saei | Characterization of Shiga toxin-producing *Escherichia coli* (STEC) in feces of healthy and diarrheic calves in Urmia region, Iran | Iran | 2010 | Diarrheic, Healthy | 124 | 4 | eaeA, hly, stx1, stx2 |
| 12 | 2012 | Barigye | Prevalence and antimicrobial susceptibility of virulent and avirulent multidrug-resistant *Escherichia coli* isolated from diarrheic neonatal calves | USA | 2010 | Diarrheic | 97 | 6 | eaeA, F41, F5, STI, stx1, stx2 |
| 13 | 2011 | Nguyen | Virulence factors in *Escherichia coli* isolated from calves with diarrhea in Vietnam | Vietnam | 2007 | Diarrheic | 345 | 10 | eaeA, F17, F41, F5, hly, LT, saa, STI, stx1, stx2 |
| 14 | 2011 | Torsein | Risk factors for calf mortality in large Swedish dairy herds | Sweden | 2006 | Unknown | 287 | 1 | F5 |
| 15 | 2011 | Pereira | Antimicrobial resistance and prevalence of virulence factor genes in fecal *Escherichia coli* of Holstein calves fed milk with and without antimicrobials | USA | 2009 | Diarrheic, Healthy | 117 | 27 | aaf, Afa, bfp, cdt, CNF, eaeA, EAST, ent, escV, fim, fyuA, hly, ibe, inv, ipa, iutA, kpsMII, LT, malX, Pap, pic, sfa, STI, stx1, stx2, traT, uidA |
| 16 | 2011 | Izzo | Prevalence of major enteric pathogens in Australian dairy calves with diarrhoea | Australia | 2008 | Diarrheic | 597 | 1 | F5 |
| 17 | 2010 | Badouei | Virulence gene profiles and intimin subtypes of Shiga toxin-producing *Escherichia coli* isolated from healthy and diarrhoeic calves | Iran | 2005* | Diarrheic, Healthy | 297 | 4 | eaeA, hly, stx1, stx2 |
| 18 | 2010 | Al-Charrakh | Prevalence of Verotoxin-Producing *Escherichia coli* (VTEC) in a survey of dairy cattle in Najaf, Iraq | Iraq | 2006 | Diarrheic | 326 | 1 | hly |
| 19 | 2010 | Bartels | Prevalence, prediction and risk factors of enteropathogens in normal and non-normal faeces of young Dutch dairy calves | Netherlands | 2007 | Diarrheic, Healthy | 424 | 1 | F5 |
| 20 | 2010 | Cho | Development of a panel of multiplex real-time polymerase chain reaction assays for simultaneous detection of major agents causing calf diarrhea in feces | USA | 2007 | Diarrheic | 243 | 1 | F5 |
| 21 | 2010 | Silverl?s | *Cryptosporidium* infection in herds with and without calf diarrhoeal problems | Sweden | 2007 | Unknown | 56 | 1 | F5 |
| 22 | 2010 | Masana | Prevalence, Characterization, and Genotypic Analysis of *Escherichia coli* O157:H7/NM from Selected Beef Exporting Abattoirs of Argentina | Argentina | 2007 | Mixed | 8 | 4 | eaeA, hly, stx1, stx2 |
| 23 | 2009 | Bardiau | Putative Adhesins of Enteropathogenic and Enterohemorrhagic *Escherichia coli* of Serogroup O26 Isolated from Humans and Cattle | USA\|Ireland\|Belgium\|France\|Japan\|Brazil | 2004* | Diarrheic, Healthy | 39 | 11 | Afa, CS31, EFA1, eibG, F17, hcp, iha, ldaE, paa, saa, toxB |
| 24 | 2009 | Ghanbarpour | Characteristics and virulence genes of *Escherichia coli* isolated from septicemic calves in southeast of Iran | Iran | 2002 | Diarrheic | 31 | 17 | Afa, cdt, CNF, CS31, eaeA, F17, F41, F5, hly, ipa, iucD, LTI, Pap, sfa, STI, stx1, stx2 |
| 25 | 2009 | Gulliksen | Enteropathogens and risk factors for diarrhea in Norwegian dairy calves | Norway | 2006 | Diarrheic | 191 | 1 | F5 |
| 26 | 2009 | Fernandez | Seasonal variation of Shiga toxin-encoding genes (stx) and detection of *E. coli* O157 in dairy cattle from Argentina | Argentina | 2006 | Healthy | 252 | 3 | eaeA, stx1, stx2 |
| 27 | 2009 | Younis | Molecular screening and risk factors of enterotoxigenic *Escherichia coli* and *Salmonella* spp. in diarrheic neonatal calves in Egypt | Egypt | 2004* | Diarrheic | 193 | 1 | F5 |
| 28 | 2008 | Gueler | Virulence Factors and Antimicrobial Susceptibility of *Escherichia coli* Isolated from Calves in Turkey | Turkey | 2004 | Diarrheic, Healthy | 120 | 7 | eaeA, F17, F41, F5, STI, stx1, stx2 |
| 29 | 2008 | Ok | The Studies on the Aetiology of Diarrhoea in Neonatal Calves and Determination of Virulence Gene Markers of *Escherichia coli* Strains by Multiplex PCR | Turkey | 2003* | Diarrheic, Healthy | 55 | 6 | eaeA, F41, F5, STI, stx1, stx2 |
| 30 | 2008 | Lee | Occurrence and characteristics of enterohemorrhagic *Escherichia coli* O26 and O111 in calves associated with diarrhea | South Korea | 2003* | Diarrheic, Healthy | 94 | 4 | eaeA, hly, stx1, stx2 |
| 31 | 2008 | Uhde | Prevalence of four enteropathogens in the faeces of young diarrhoeic dairy calves in Switzerland | Switzerland | 2006 | Diarrheic | 147 | 1 | F5 |
| 32 | 2008 | Arya | Serogroups, Atypical Biochemical Characters, Colicinogeny and Antibiotic Resistance Pattern of Shiga Toxin-producing *Escherichia coli* Isolated from Diarrhoeic Calves in Gujarat, India | India | 2004 | Diarrheic | 90 | 3 | eaeA, stx1, stx2 |
| 33 | 2008 | Cristancho | *Escherichia coli* O157:H7 and other Shiga toxin-producing *E. coli* in white veal calves | Canada | 2003* | Mixed | 1025 | 5 | eaeA, hly, stx, stx1, stx2 |
| 34 | 2008 | Evans | Cefixime–tellurite rhamnose MacConkey agar for isolation of Vero cytotoxin-producing *Escherichia coli* serogroup O26 from Scottish cattle and sheep faeces | UK | 2004 | Mixed | 112 | 4 | eaeA, hly, stx1, stx2 |
| 35 | 2007 | Wani | Variants of eae and stx genes of atypical enteropathogenic *Escherichia coli* and non-O157 Shiga toxin-producing *Escherichia coli* from calves | India | 2004 | Diarrheic, Healthy | 187 | 6 | bfp, eaeA, EAF, hly, stx1, stx2 |
| 36 | 2007 | Aidar-Ugrinovich | Serotypes, virulence genes, and intimin types of Shiga toxin-producing *Escherichia coli* (STEC) and enteropathogenic *E. coli* (EPEC) isolated from calves in Sao Paulo, Brazil | Brazil | 2002* | Diarrheic, Healthy | 70 | 7 | bfp, eaeA, EAF, hly, saa, stx1, stx2 |
| 37 | 2007 | Thompson | Genotypes and subtypes of *Cryptosporidium* spp. in neonatal calves in Northern Ireland | Ireland | 2002 | Diarrheic | 421 | 1 | F5 |
| 38 | 2006 | Manna | Occurrence, virulence characteristics and antimicrobial resistance of *Escherichia coli* O157 in slaughtered cattle and diarrhoeic calves in West Bengal, India | India | 2003 | Diarrheic | 11 | 3 | hly, stx1, stx2 |
| 39 | 2006 | Uber | Enteroaggregative *Escherichia coli* from humans and animals differ in major phenotypical traits and virulence genes | Brazil | 2001* | Diarrheic | 16 | 7 | aaf, aap, EAST, irp, pet, pic, shf |
| 40 | 2006 | Cho | Prevalence of shiga toxin-encoding bacteria and shiga toxin-producing *Escherichia coli* isolates from dairy farms and county fairs | USA | 2002 | Mixed | 385 | 8 | eaeA, esp, etpD, hly, katP, saa, stx1, stx2 |
| 41 | 2006 | Cookson | Serotypes and analysis of distribution of Shiga toxinproducing *Escherichia coli* from cattle and sheep in the lower North Island, New Zealand | New Zealand | 2002 | Healthy | 91 | 3 | eaeA, stx1, stx2 |
| 42 | 2006 | Donaldson | Molecular Epidemiology of Ceftiofur-Resistant *Escherichia coli* Isolates from Dairy Calves | USA | 2003 | Healthy | 122 | 7 | CNF, CS31, F1845, F5, STI, stx1, stx2 |
| 43 | 2005 | Orden | Detection of the *saa* gene in verotoxin-producing *Escherichia coli* from ruminants | Spain | 2000* | Diarrheic | 17 | 5 | eaeA, hly, saa, stx1, stx2 |
| 44 | 2005 | Das | Dairy farm investigation on Shiga toxin-producing *Escherichia coli* (STEC) in Kolkata, India with emphasis on molecular characterization | India | 2002 | Healthy | 13 | 5 | eaeA, etpD, hly, stx1, stx2 |
| 45 | 2004 | Pearce | Temporal Shedding Patterns and Virulence Factors of *Escherichia coli* Serogroups O26, O103, O111, O145, and O157 in a Cohort of Beef Calves and Their Dams | UK | 2001 | Mixed | 157 | 4 | eaeA, hly, stx1, stx2 |
| 46 | 2004 | Kang | Occurrence and characteristics of enterohemorrhagic *Escherichia coli* O157 in calves associated with diarrhoea | South Korea | 2001 | Diarrheic, Healthy | 31 | 4 | eaeA, hly, stx1, stx2 |
| 47 | 2004 | Mercado | Non-O157 Shiga Toxin-producing *Escherichia coli* Isolated from Diarrhoeic Calves in Argentina | Argentina | 1998 | Diarrheic | 15 | 5 | bfp, eaeA, hly, stx1, stx2 |
| 48 | 2004 | Ewers | Molecular characteristics of *Escherichia coli* serogroup O78 strains isolated from diarrheal cases in bovines urge further investigations on their zoonotic potential | Germany | 1998 | Diarrheic | 15 | 16 | cdt, CNF, eaeA, EAST, esp, F41, F5, fyuA, hly, ipa, irp, iucD, LTI, STI, stx, tia |
| 49 | 2004 | Achá | Studies on Calf Diarrhoea in Mozambique: Prevalence of Bacterial Pathogens | Mozambique | 1994 | Diarrheic, Healthy | 143 | 3 | F5, LT, STI |
| 50 | 2004 | Shaw | Shedding Patterns of Verocytotoxin-Producing *Escherichia coli* Strains in a Cohort of Calves and Their Dams on a Scottish Beef Farm | UK | 2002 | Unknown | 130 | 4 | eaeA, hly, stx1, stx2 |
| 51 | 2004 | Sisti | Isolation of *Escherichia coli* O157 from human and bovine faeces in the Urbino area, Italy | Italy | 1999* | Healthy | 1 | 3 | eaeA, stx1, stx2 |
| 52 | 2003 | Kobayashi | Prevalence and Characteristics of *eae*-Positive *Escherichia coli* from Healthy Cattle in Japan | Japan | 2001 | Healthy | 142 | 3 | eaeA, stx1, stx2 |
| 53 | 2003 | Mercado | Occurrence and Characteristics of CS31A Antigen-Producing *Escherichia coli* in Calves with Diarrhoea and Septicaemia in Argentina | Argentina | 1998* | Diarrheic | 100 | 4 | CS31, F17, F41, F5 |
| 54 | 2003 | Wani | Isolation and characterization of Shiga toxin-producing *Escherichia coli* (STEC) and enteropathogenic *Escherichia coli* (EPEC) from calves and lambs with diarrhoea in India | India | 2001 | Diarrheic | 113 | 4 | eaeA, hly, stx1, stx2 |
| 55 | 2003 | Leomil | Frequency of Shiga toxin-producing *Escherichia coli* (STEC) isolates among diarrheic and non-diarrheic calves in Brazil | Brazil | 1998* | Diarrheic, Healthy | 24 | 4 | eaeA, hly, stx1, stx2 |
| 56 | 2003 | Girardeau | Extended Virulence Genotype of Pathogenic *Escherichia coli* Isolates Carrying the afa-8 Operon: Evidence of Similarities between Isolates from Humans and Animals with Extraintestinal Infections | Belgium\|France\|Spain | 1998* | Diarrheic | 42 | 16 | Afa, CNF, CS31, EAST, F17, hly, HPI, iha, iroN, iutA, kpsMII, malX, modD, Pap, sfa, stx |
| 57 | 2003 | Bjoerkman | *Cryptosporidium parvum* and *Giardia intestinalis* in Calf Diarrhoea in Sweden | Sweden | 1998 | Diarrheic, Healthy | 270 | 1 | F5 |
| 58 | 2003 | Chinen | Isolation and characterisation of Shiga toxin-producing *Escherichia coli* O157:H7 from calves in Argentina | Argentina | 1998 | Diarrheic, Healthy | 2 | 4 | eaeA, hly, stx1, stx2 |
| 59 | 2002 | Orden | Verotoxin-producing *Escherichia coli* (VTEC), enteropathogenic *E. coli* (EPEC) and necrotoxigenic *E. coli* (NTEC) isolated from healthy cattle in Spain | Spain | 1996 | Healthy | 101 | 4 | CNF, eaeA, stx1, stx2 |
| 60 | 2002 | Tutenel | Isolation and molecular characterization of *Escherichia coli* O157 isolated from cattle, pigs and chickens at slaughter | Belgium | 2000 | Healthy | 25 | 4 | eaeA, hly, stx1, stx2 |
| 61 | 2002 | Giammanco | Characterization of Shiga Toxin-Producing *Escherichia coli* O157:H7 Isolated in Italy and in France | Italy\|France | 1997* | Healthy | 37 | 4 | eaeA, hly, stx1, stx2 |
| 62 | 2001 | Fecteau | Virulence factors in *Escherichia coli* isolated from the blood of bacteremic neonatal calves | USA | 1992 | Diarrheic | 25 | 13 | aerobactin, Afa, CNF, CS31, eaeA, F17, hly, LTI, Pap, STI, STII, stx1, stx2 |
| 63 | 2001 | Osek | Prevalence of *eae* and Shiga Toxin Genes among *Escherichia coli* Strains Isolated from Healthy Calves | Poland | 1996* | Healthy | 390 | 9 | eaeA, F17, F41, F5, LTI, STI, STII, stx1, stx2 |
| 64 | 2001 | Osek | Characterization of Necrotoxigenic *Escherichia coli* (NTEC) Strains Isolated from Healthy Calves in Poland | Poland | 1996* | Healthy | 396 | 10 | CNF, eaeA, F17, F41, F5, LTI, STI, STII, stx1, stx2 |
| 65 | 2001 | Van Bost | Characteristics of necrotoxigenic *Escherichia coli* isolated from septicemic and diarrheic calves between 1958 and 1970. | Belgium | 1964 | Diarrheic | 864 | 6 | Afa, cdt, CNF, F17, Pap, sfa |
| 66 | 2001 | Cobbold | Characterisation and clonal relationships of Shiga-toxigenic *Escherichia coli* (STEC) isolated from Australian dairy cattle | Australia | 1996* | Healthy, Mixed | 95 | 4 | eaeA, hly, stx1, stx2 |
| 67 | 2000 | Shinagawa | Frequency of Shiga toxin-producing *Escherichia coli* in cattle at a breeding farm and at a slaughterhouse in Japan | Japan | 1998 | Mixed | 204 | 2 | stx1, stx2 |
| 68 | 2000 | Bertin | Epidemiological Study of *pap* Genes among Diarrheagenic or Septicemic *Escherichia coli* Strains Producing CS31A and F17 Adhesins and Characterization of Pap31A Fimbriae | France\|Belgium | 1995* | Diarrheic | 118 | 3 | CS31, F17, Pap |
| 69 | 2000 | Osek | Characterization of shiga toxin-producing *Escherichia coli* strains isolated from calves in Poland | Poland | 1995* | Diarrheic | 190 | 12 | CNF, eaeA, F17, F41, F5, hly, LTI, Pap, STI, STII, stx1, stx2 |
| 70 | 2000 | Aidar | Subtypes of intimin among non-toxigenic *Escherichia coli* from diarrheic calves in Brazil | Brazil | 1995* | Diarrheic | 105 | 1 | eaeA |
| 71 | 2000 | Garcia | Rotavirus and concurrent infections with other enteropathogens in neonatal diarrheic dairy calves in Spain | Spain | 1994 | Diarrheic | 218 | 1 | F5 |
| 72 | 1999 | Bonardi | Isolation of Verocytotoxin-producing *Escherichia coli* O157 : H7 from cattle at slaughter in Italy | Italy | 1994* | Healthy | 90 | 4 | eaeA, stx, stx1, stx2 |
| 73 | 1999 | China | Heterogeneity of the *eae* genes in attaching/effacing *Escherichia coli* from cattle: comparison with human strains | USA | 1994* | Diarrheic | 191 | 1 | eaeA |
| 74 | 1999 | de la Fuente | *Cryptosporidium* and concurrent infections with otherm major enterophatogens in 1 to 30-day-old diarrheic dairy calves in central Spain | Spain | 1994 | Diarrheic | 114 | 1 | F5 |
| 75 | 1999 | Holland | Characterization of *eae Escherichia coli* isolated from healthy and diarrheic calves | USA | 1994* | Diarrheic, Healthy | 215 | 3 | eaeA, hly, stx |
| 76 | 1998 | Wieler | Virulence Properties of Shiga Toxin-Producing *Escherichia coli* (STEC) Strains of Serogroup O118, a Major Group of STEC Pathogens in Calves | Germany | 1992 | Diarrheic, Healthy | 42 | 3 | hly, stx1, stx2 |
| 77 | 1998 | Orden | Verotoxin-producing *Escherichia coli* (VTEC) and eae-positive non-VTEC in 1±30-days-old diarrhoeic dairy calves | Spain | 1993* | Diarrheic | 861 | 4 | eaeA, esp, stx1, stx2 |
| 78 | 1998 | China | Prevalence and molecular typing of attaching and effacing *Escherichia coli* among calf populations in Belgium | Belgium | 1993* | Diarrheic, Healthy | 7708 | 3 | eaeA, stx1, stx2 |
| 79 | 1998 | Blanco | Prevalence and characteristics of necrotoxigenic *Escherichia coli* CNF1+ and CNF2+ in healthy cattle | Spain | 1994 | Healthy | 71 | 1 | CNF |
| 80 | 1998 | Contrepois | A study of relationships among F17 a producing enterotoxigenic and non-enterotoxigenic *Escherichia coli* strains isolated from diarrheic calves | France\|Belgium | 1993* | Diarrheic | 41 | 2 | F17, F5 |
| 81 | 1998 | de la Fuente | Proportional morbidity rates of enteropathogens among diarrheic dairy calves in central Spain | Spain | 1994 | Diarrheic | 218 | 1 | F5 |
| 82 | 1997 | Saridakis | Virulence properties of *Escherichia coli* strains belonging to enteropathogenic (EPEC) serogroups isolated from calves with diarrhea | Brazil | 1993* | Diarrheic | 19 | 2 | eaeA, EAF |
| 83 | 1997 | Blanco | Distribution and characterization of faecal verotoxin-producing *Escherichia coli* (VTEC) isolated from healthy cattle | Spain | 1993* | Healthy | 33 | 3 | eaeA, stx1, stx2 |
| 84 | 1997 | Rahn | Persistence of *Escherichia coli* O157:H7 in dairy cattle and the dairy farm environment | Canada | 1993* | Mixed | 115 | 1 | stx |
| 85 | 1997 | Mechie | A fifteen month study of *Escherichia coli* O157:H7 in a dairy herd | UK | 1994 | Mixed | 44 | 2 | stx1, stx2 |
| 86 | 1996 | China | Typing of Bovine Attaching and Effacing *Escherichia coli* by Multiplex In Vitro Amplification of Virulence-Associated Genes | Unknown | 1992* | Diarrheic | 30 | 3 | eaeA, stx1, stx2 |
| 87 | 1996 | Wieler | Shiga Toxin-Producing *Escherichia coli* Strains from Bovines: Association of Adhesion with Carriage of *eae* and Other Genes | Germany\|Belgium | 1992 | Diarrheic | 174 | 5 | bfp, eaeA, esp, stx1, stx2 |
| 88 | 1996 | Sandhu | Prevalence of the *eaeA* gene in verotoxigenic *Escherichia coli* strains from dairy cattle in Southwest Ontario | Canada | 1992* | Healthy | 592 | 3 | eaeA, stx1, stx2 |
| 89 | 1996 | Blanco | Prevalence and characteristics of *Escherichia coli* serotype 0157:H7 and other verotoxin-producing E. coli in healthy cattle | Spain | 1994 | Healthy | 23 | 3 | eaeA, stx1, stx2 |
| 90 | 1995 | Ganaba | Importance of *Escherichia coli* in Young Beef Calves from Northwestern Quebec | Canada | 1991 | Unknown, Mixed | 400 | 4 | F165, F41, F5, STI |
| 91 | 1995 | Zhao | Prevalence of Enterohemorrhagic *Escherichia coli* O157:H7 in a Survey of Dairy Herds | USA | 1993 | Mixed | 31 | 2 | stx1, stx2 |
| 92 | 1994 | Shin | Hybridization of clinical *Escherichia coli* isolates from calves and piglets in New York State with gene probes for enterotoxins (STaP, STb, LT), Shiga-like toxins (SLT- 1, SLT-II) and adhesion factors (K88, K99, F41,987P) | USA | 1986 | Diarrheic | 666 | 9 | F4, F41, F5, F6, LT, STI, STII, stx1, stx2 |
| 93 | 1994 | Blanco | Genes coding for Shiga-like toxins in bovine verotoxin-producing *Escherichia coli* (VTEC) strains belonging to different O:K:H serotypes | Spain | 1986 | Diarrheic, Healthy | 46 | 6 | LTI, LTII, STI, STII, stx1, stx2 |
| 94 | 1992 | Wilson | Distribution and characteristics of verocytotoxigenic *Escherichia coli* isolated from Ontario dairy cattle | Canada | 1988 | Mixed | 659 | 1 | stx |
| 95 | 1991 | Oswald | Virulence Factors Associated with Cytotoxic Necrotizing Factor Type Two in Bovine Diarrheic and Septicemic Strains of *Escherichia coli* | Unknown | 1987* | Diarrheic | 36 | 11 | CNF, F17, F4, F41, F5, LTI, LTII, STI, STII, stx1, stx2 |
| 96 | 1990 | Samadpour | Evaluation of DNA Probes for Detection of Shiga-Like-Toxin-Producing *Escherichia coli* in Food and Calf Fecal Samples | USA | 1986* | Mixed | 28 | 1 | stx |
| 97 | 1990 | Scotland | Properties of Strains of *Escherichia coli* 026:811 in Relation to Their Enteropathogenic or Enterohemorrhagic Classification | UK | 1986* | Diarrheic | 8 | 3 | hly, stx1, stx2 |
| 98 | 1989 | Janke | Attaching and effacing *Escherichia coli* infections in calves, pigs, lambs, and dogs | USA | 1985* | Diarrheic | 14 | 1 | stx |
| 99 | 1987 | Raybould | Monoclonal Antibody Passive Hemagglutination and Capture Enzyme-Linked Immunosorbent Assays for Direct Detection and Quantitation of F41 and K99 Fimbrial Antigens in Enterotoxigenic *Escherichia coli* | Canada | 1983* | Diarrheic, Healthy | 10 | 2 | F41, F5 |
| 100 | 1987 | de Visser | Enteric infections in veal calves: a longitudinal study on four veal calf units | Netherlands | 1983 | Diarrheic | 45 | 1 | F5 |
| 101 | 1986 | Waltner-Toews | An Epidemiological Study of Selected Calf Pathogens on Holstein Dairy Farms in Southwestern Ontario | Canada | 1982 | Diarrheic, Healthy | 86 | 1 | F5 |
| 102 | 1985 | Harnett | Enterotoxin Plasmids in Bovine and Porcine Enterotoxigenic *Escherichia coli* of O Groups 9, 20, 64 and 101 | Canada | 1981* | Diarrheic | 3 | 1 | F5 |
| 103 | 1984 | Mills | Monoclonal Antibody Enzyme-Linked Immunosorbent Assay for Identification of K99-Positive *Escherichia coli* Isolates from Calves | Unknown | 1980* | Diarrheic | 251 | 1 | F5 |
| 104 | 1979 | Ellens | Detection of the K99 antigen of *Escherichia coli* in calf faeces by enzyme-linked immunosorbent assay (ELISA) | Netherlands | 1975* | Mixed | 88 | 1 | F5 |
| 105 | 1976 | Myers | Occurrence and Characteristics of Enterotoxigenic *Escherichia coli* Isolated from Calves with Diarrhea | USA | 1975 | Diarrheic | 35 | 1 | F5 |
| 106 | 1976 | Guinée | Detection of the K99 Antigen by Means of Agglutination and Immunoelectrophoresis in *Escherichia coli* Isolates from Calves and Its Correlation with Enterotoxigenicity | Netherlands\|USA\|UK | 1972* | Diarrheic | 94 | 1 | F5 |

**Supplementary Table 3. Methods for *E. coli* characterization.**

| Method name | Number of publications |
| --- | --- |
| PCR | 67 |
| Colony hybridization | 22 |
| ELISA | 13 |
| Slide agglutination | 8 |
| IFAT | 1 |
| Serotyping | 54 |
| Antimicrobial susceptibility tests | 19 |
| Clonal relationships | 15 |

**Supplementary Table 4. Comparison of study groups (mixed and unknown).**

| VAF name | Group | Number of publications | Isolates positive | Isolates tested | Proportion of positives |
| --- | --- | --- | --- | --- | --- |
| bfp | Mixed | 1 | 0 | 48 | 0.0000 |
| eaeA | Mixed | 2 | 70 | 178 | 0.3933 |
| EAST | Mixed | 1 | 35 | 142 | 0.2465 |
| esp | Mixed | 1 | 18 | 96 | 0.1875 |
| etpD | Mixed | 1 | 6 | 48 | 0.1250 |
| F165 | Mixed | 1 | 21 | 373 | 0.0563 |
| F18 | Mixed | 1 | 0 | 94 | 0.0000 |
| F4 | Mixed | 1 | 0 | 94 | 0.0000 |
| F41 | Mixed | 2 | 77 | 467 | 0.1649 |
| F5 | Mixed | 4 | 2 | 810 | 0.0025 |
| F6 | Mixed | 1 | 0 | 94 | 0.0000 |
| fim | Mixed | 1 | 43 | 48 | 0.8958 |
| fyuA | Mixed | 1 | 17 | 48 | 0.3542 |
| hly | Mixed | 2 | 104 | 226 | 0.4602 |
| katP | Mixed | 1 | 6 | 48 | 0.1250 |
| LT | Mixed | 1 | 0 | 94 | 0.0000 |
| STI | Mixed | 2 | 13 | 467 | 0.0278 |
| STII | Mixed | 1 | 12 | 94 | 0.1277 |
| stx1 | Mixed | 2 | 61 | 178 | 0.3427 |
| stx2 | Mixed | 2 | 108 | 272 | 0.3971 |
| eaeA | Unknown | 7 | 327 | 450 | 0.7267 |
| esp | Unknown | 1 | 10 | 21 | 0.4762 |
| etpD | Unknown | 1 | 3 | 21 | 0.1429 |
| F165 | Unknown | 1 | 3 | 27 | 0.1111 |
| F41 | Unknown | 1 | 15 | 27 | 0.5556 |
| F5 | Unknown | 2 | 32 | 115 | 0.2783 |
| hly | Unknown | 7 | 323 | 450 | 0.7178 |
| katP | Unknown | 1 | 6 | 21 | 0.2857 |
| saa | Unknown | 1 | 1 | 21 | 0.0476 |
| stx | Unknown | 4 | 921 | 1827 | 0.5041 |
| stx1 | Unknown | 10 | 413 | 1093 | 0.3779 |
| stx2 | Unknown | 10 | 274 | 1093 | 0.2507 |

**Supplementary Table 5. Biological assays.**

| Assay name | Number of publications | Phenotype investigated |
| --- | --- | --- |
| Vero cytotoxicity assay | 27 | Stx production, adhesion patterns |
| HEp-2 adhesion assay | 9 | Adhesion patterns |
| Infant mouse assay | 6 | Enterotoxin production |
| Ligated intestinal loops | 4 | Enterotoxin production |
| HeLa cell assay | 3 | CPE |
| Calf villi assay | 2 | Adhesion |
| MBDK assay | 1 | Adhesion |

## Suplementary Figures


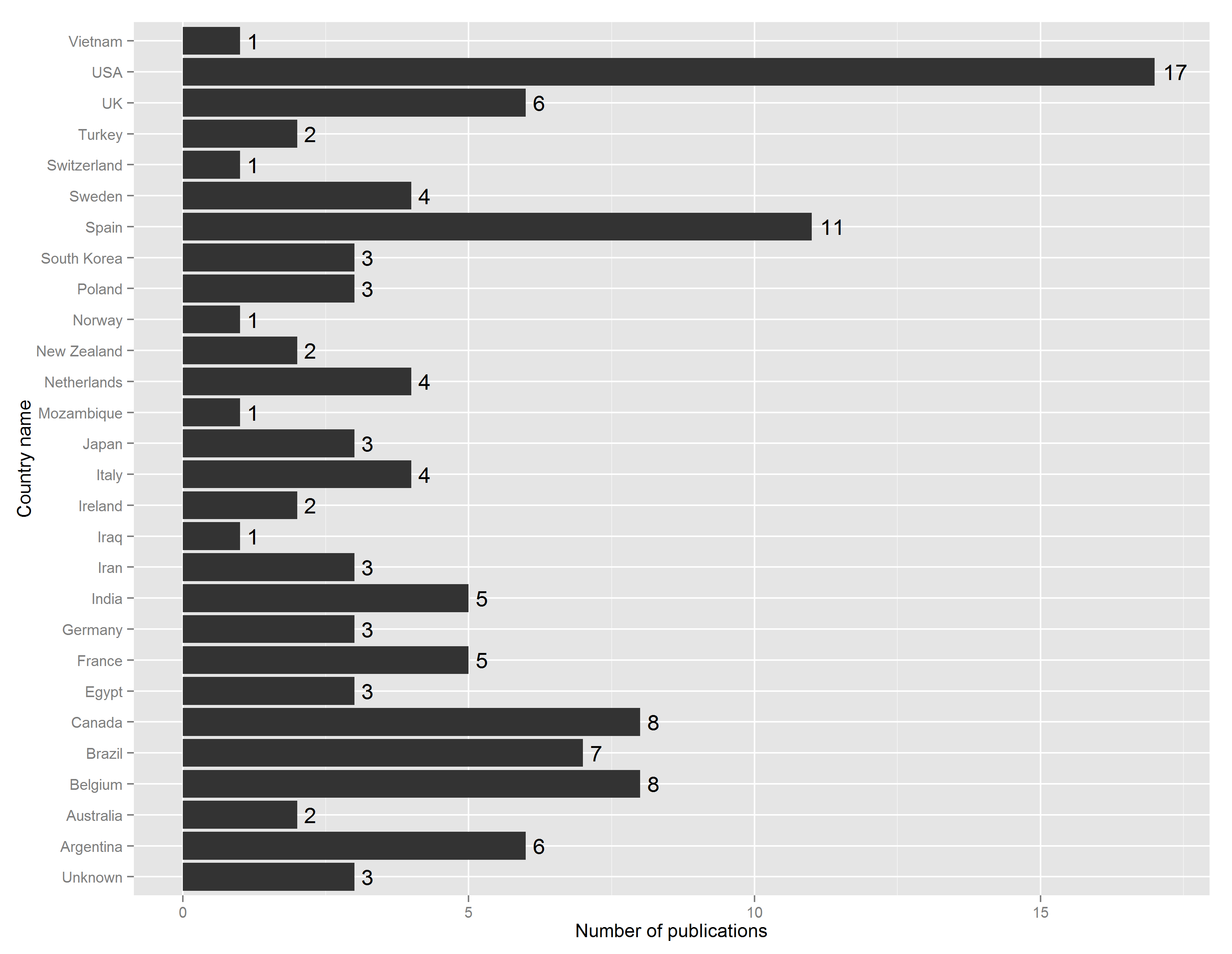


**Supplementary Fig. 1 Number of publications according to study country.** Unknown: unknown source of isolates


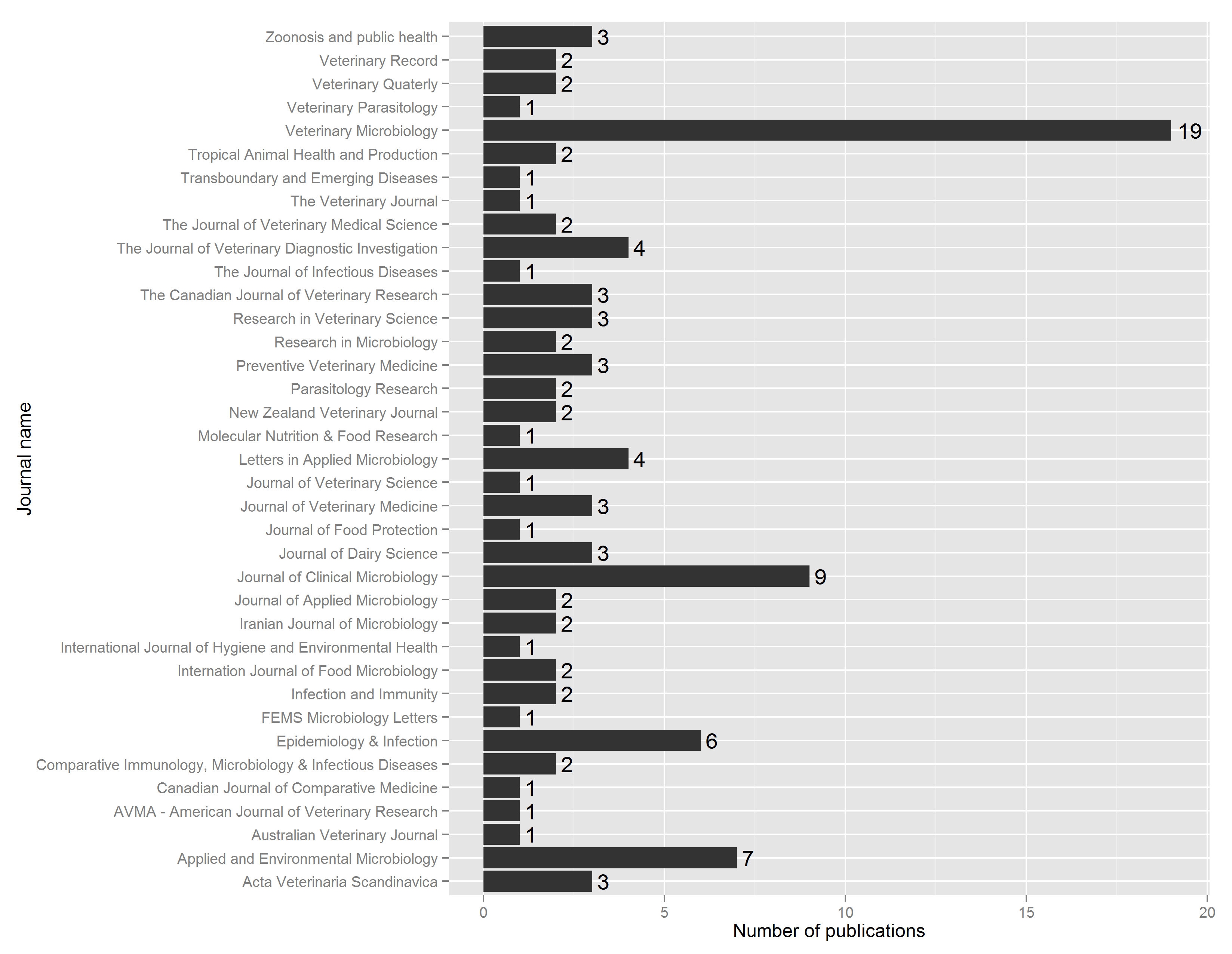


**Supplementary Fig. 2 Number of publications according to publishing journal**


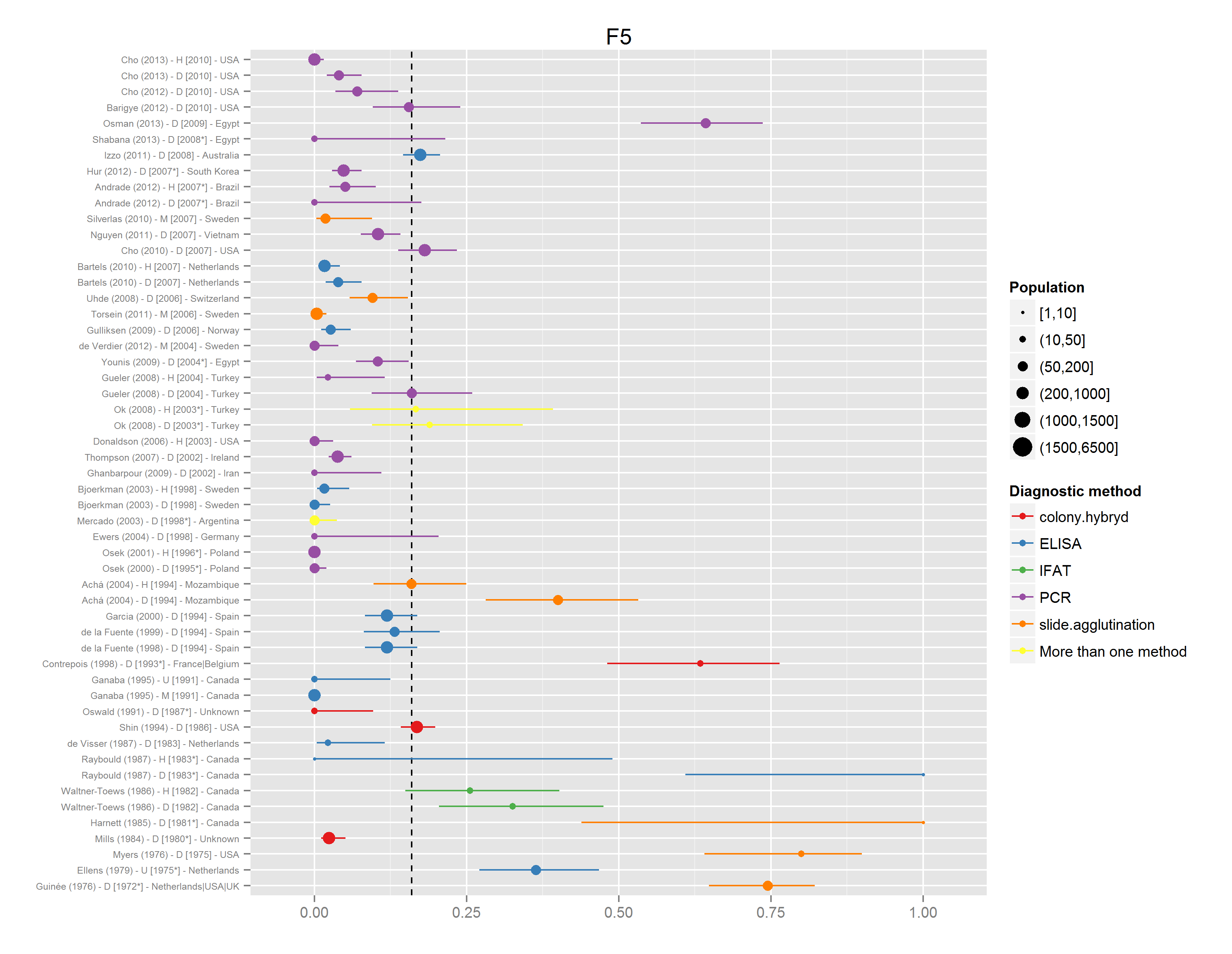


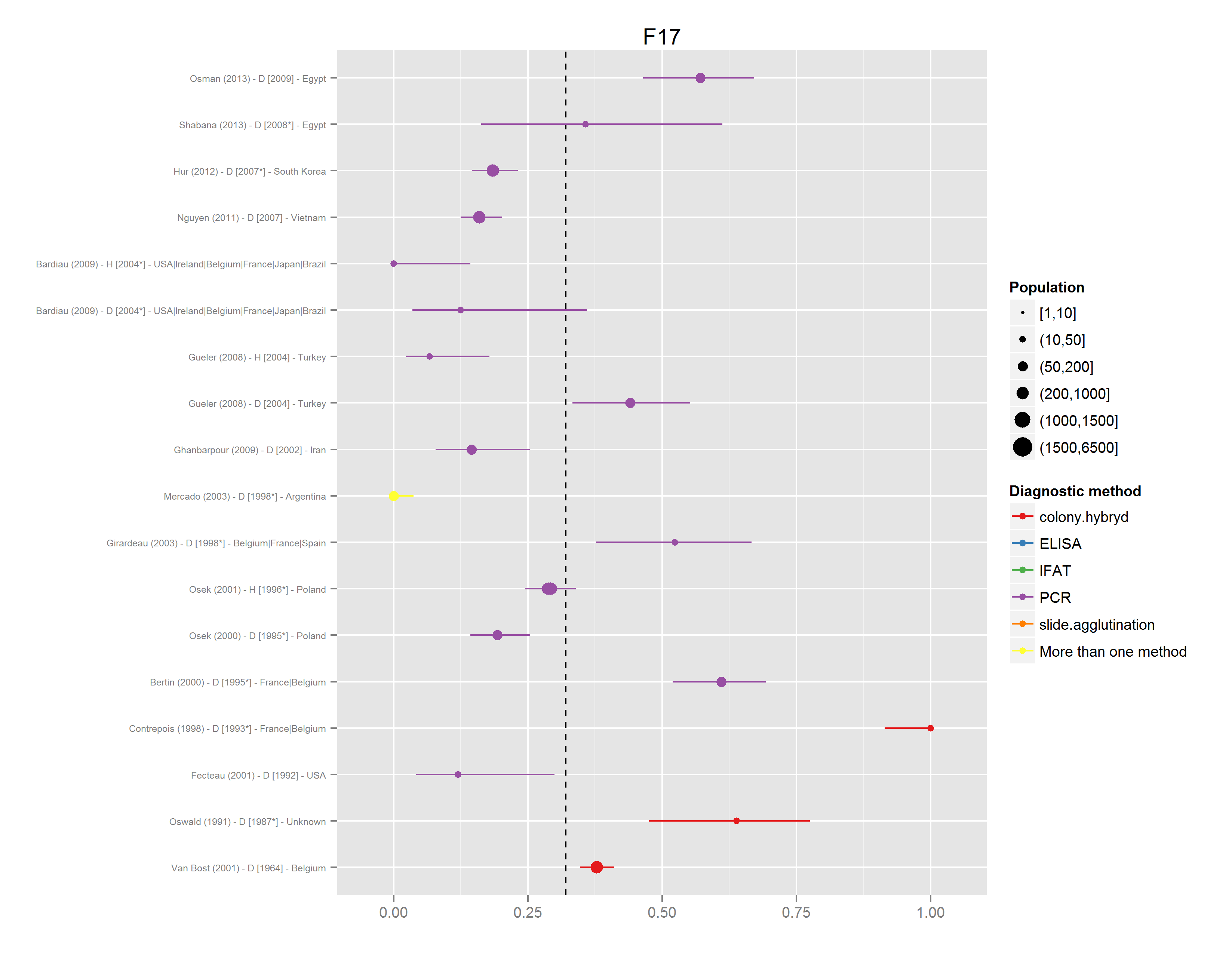


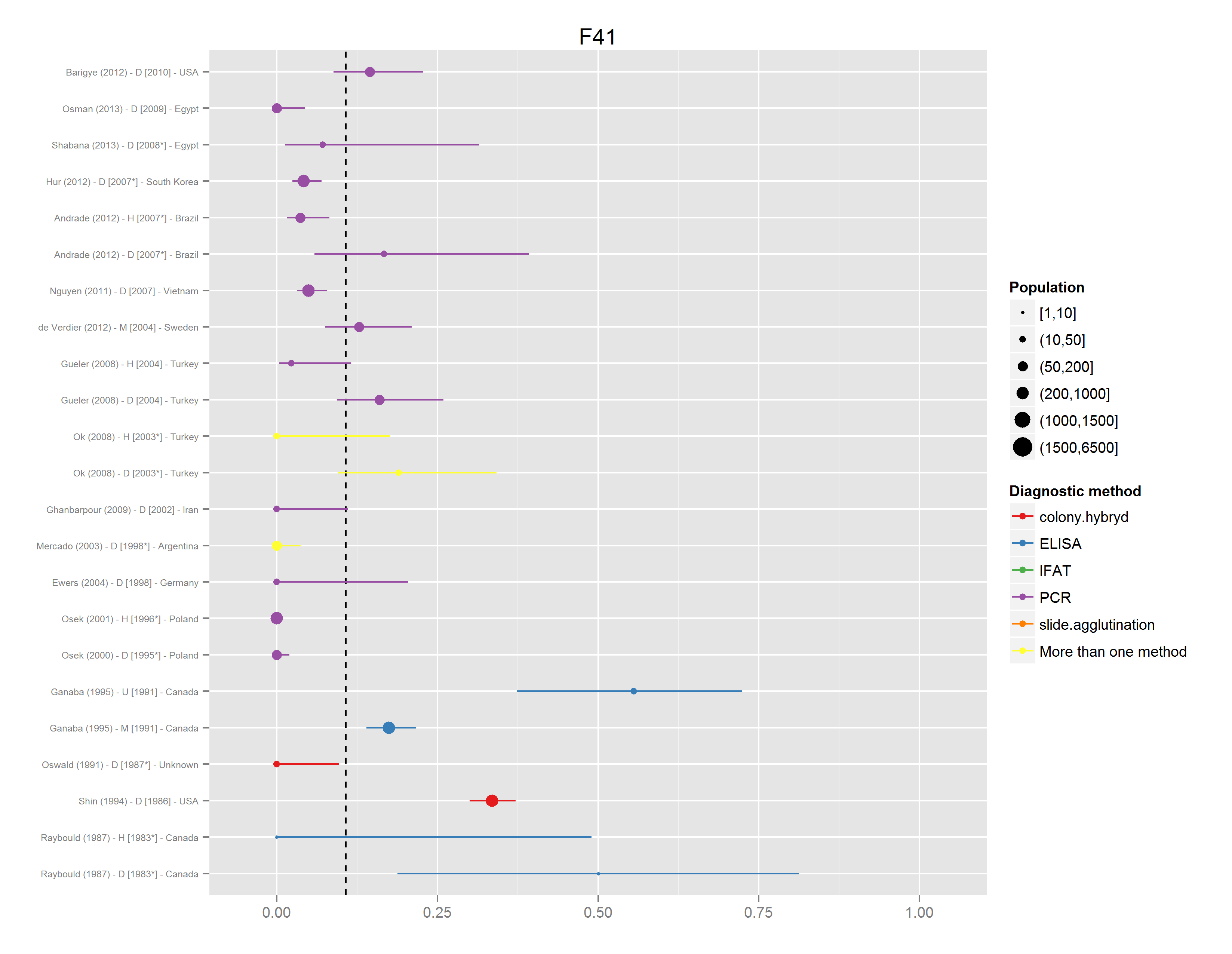


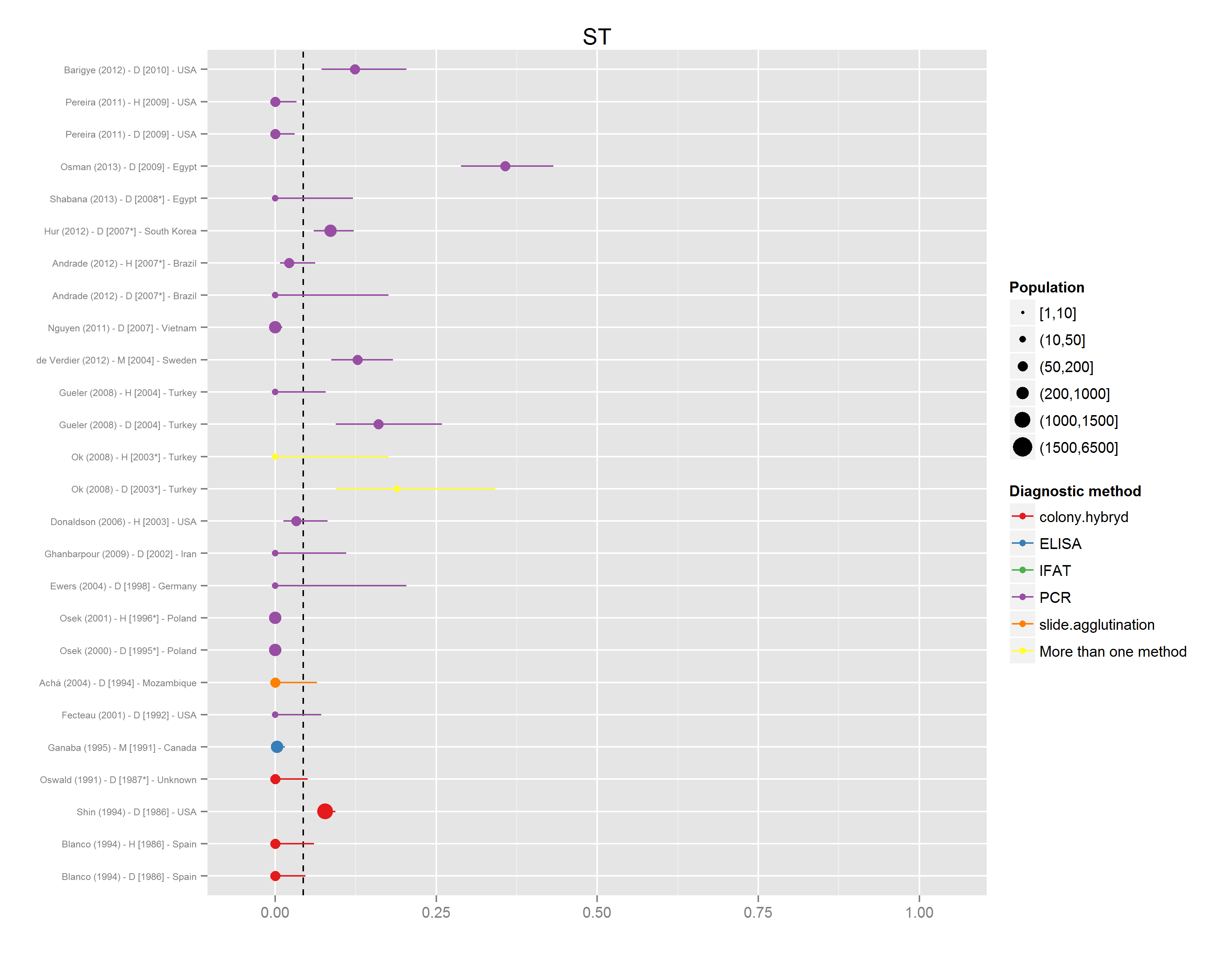


**Supplementary Fig. 3** Prevalence of ETEC VAFs in the manuscripts under review. The dashed vertical lines represent the mean prevalence of the given VAF for all studies. Dashed vertical lines represent prevalence of a VAF. Labels contain information about the first author of the study, the year of publication, the health status of animals (H – healthy, D – diarrheic, M – mixed, U – unknown), year of isolation (with the asterisk if isolation date was extrapolated) and country of isolates' origin (NA means no information about country). Color of the points indicates diagnostic method(s) used in a study. Horizontal lines represent the 0.95 confidence intervals. Point size represents number of isolates with given VAF in single study. Data were sorted according to the year of isolation


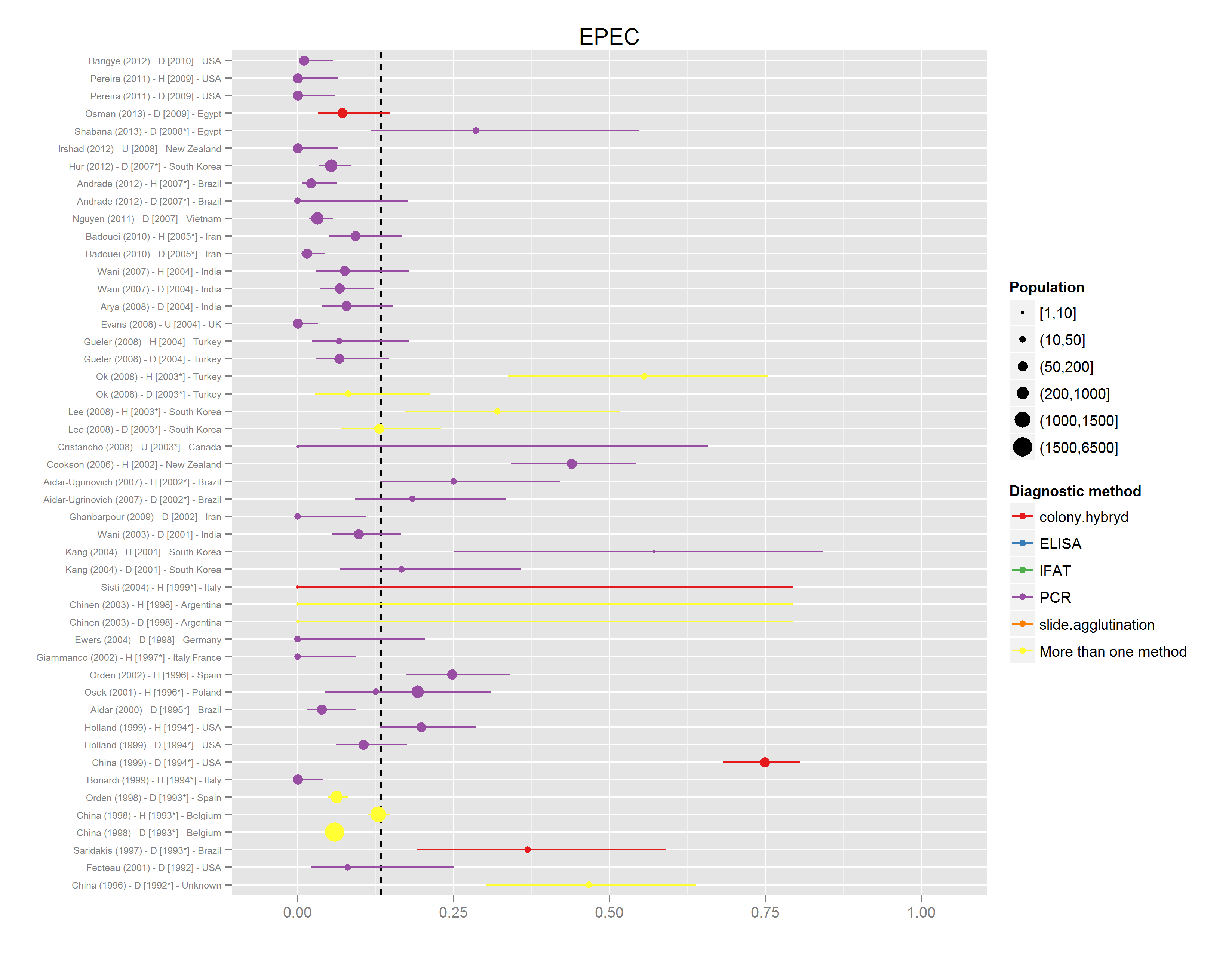


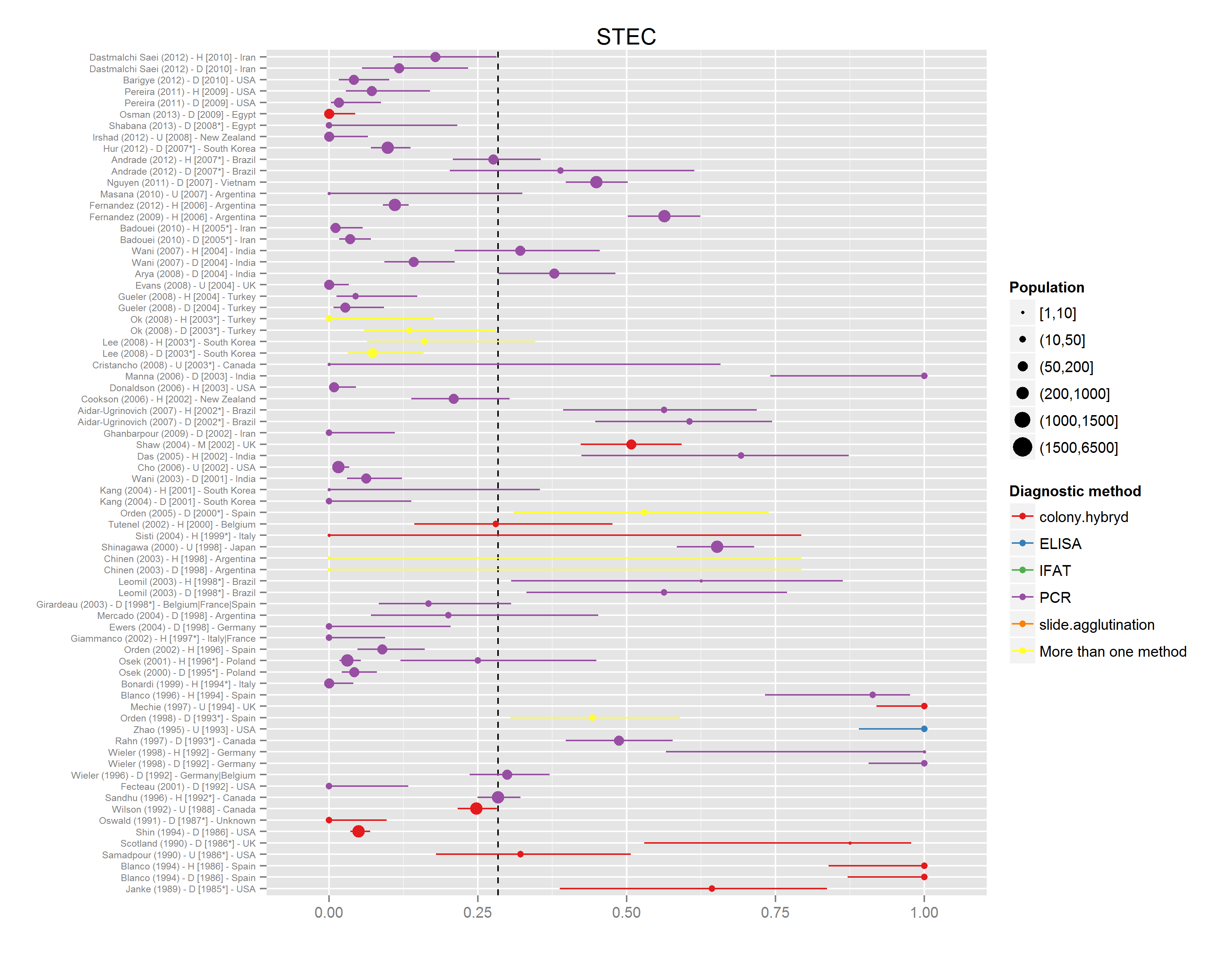


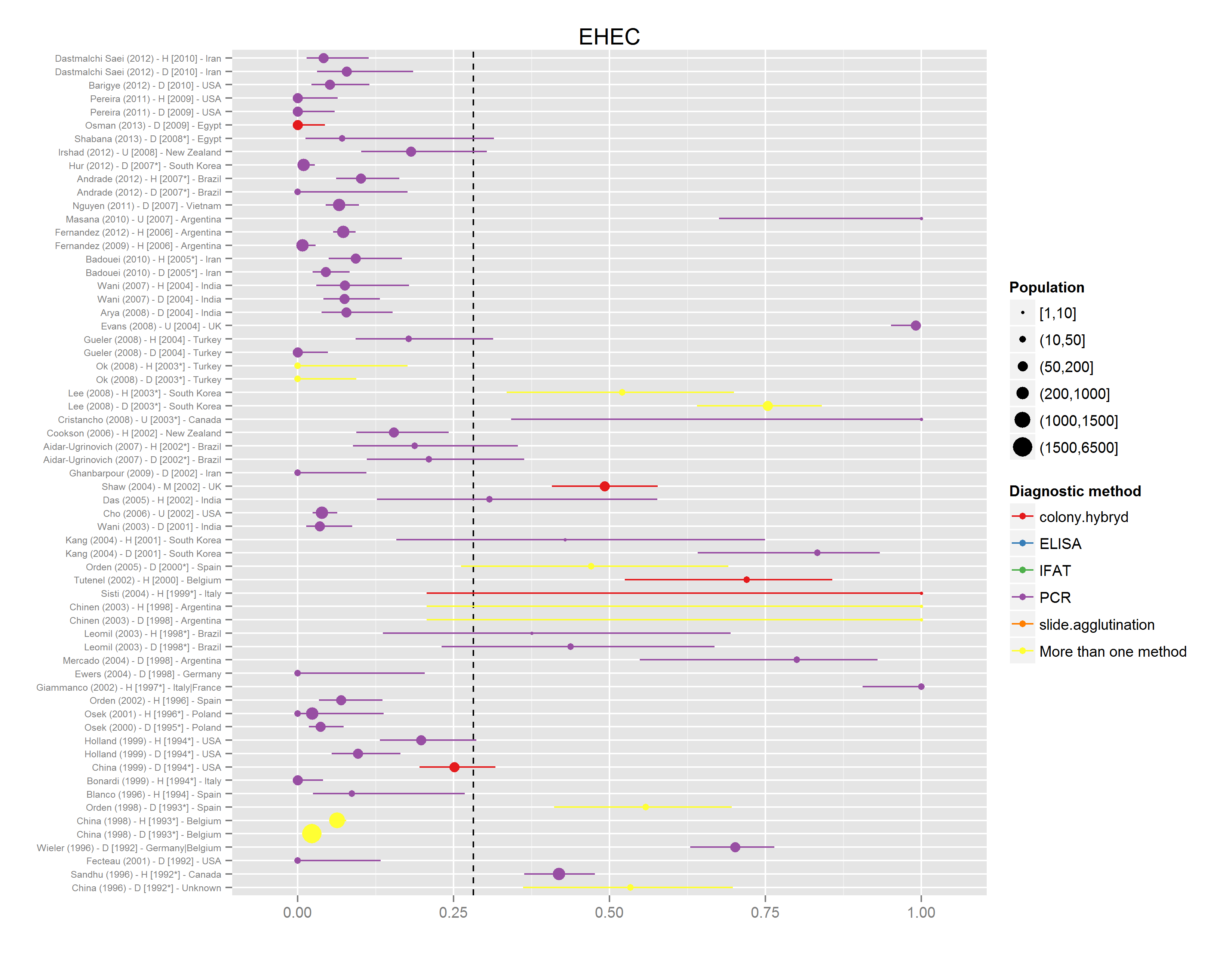


**Supplementary Fig. 4 Prevalence of STEC, EPEC, EHEC in the manuscripts.** The dashed vertical lines represent the mean prevalence of the given pathotype of all studies. Labels contain information about the first author of the study, the year of publication, the health status of animals (H – healthy, D – diarrheic, M – mixed, U – unknown), year of isolation (with the asterisk if isolation date was extrapolated) and country of isolates' origin (NA means no information about country). Horizontal lines represent the 0.95 confidence intervals. Color of the points indicates the diagnostic method(s) used in a study. Point size represents number of isolates for a pathotype


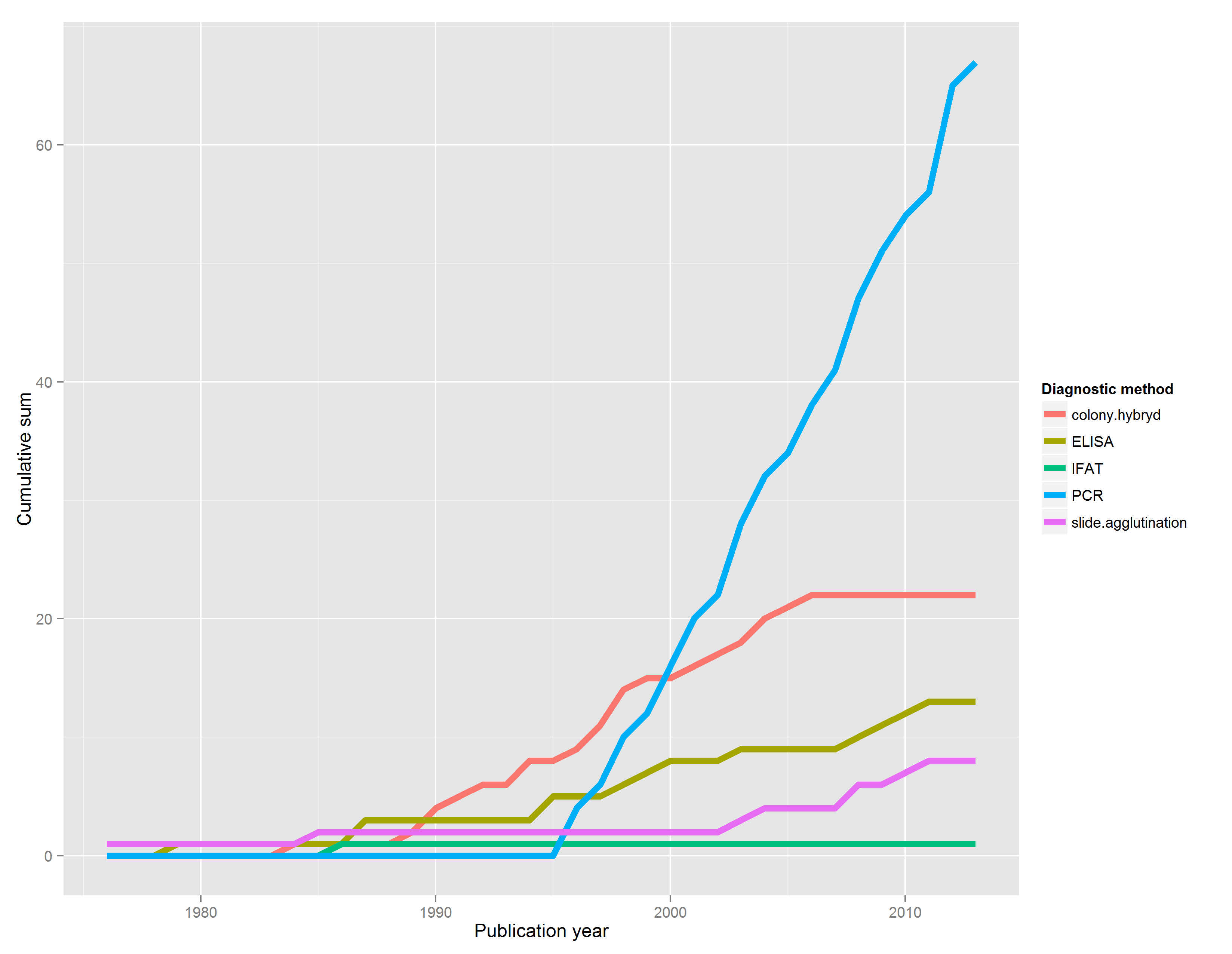


**Supplementary Fig. 5** Line chart showing the cumulative sum of studies utilizing a given diagnostic method and the year of publication
